# Getting a Grip on the Undrugged: Targeting β‐Catenin with Fragment‐Based Methods

**DOI:** 10.1002/cmdc.202000839

**Published:** 2021-01-14

**Authors:** Dirk Kessler, Moriz Mayer, Stephan K. Zahn, Markus Zeeb, Simon Wöhrle, Andreas Bergner, Jens Bruchhaus, Tuncay Ciftci, Georg Dahmann, Maike Dettling, Sandra Döbel, Julian E. Fuchs, Leonhard Geist, Wolfgang Hela, Christiane Kofink, Roland Kousek, Franziska Moser, Teresa Puchner, Klaus Rumpel, Maximilian Scharnweber, Patrick Werni, Bernhard Wolkerstorfer, Dennis Breitsprecher, Philipp Baaske, Mark Pearson, Darryl B. McConnell, Jark Böttcher

**Affiliations:** ^1^ Boehringer Ingelheim RCV GmbH & Co KG Dr.-Boehringer-Gasse 5–11 1121 Vienna Austria; ^2^ Boehringer Ingelheim Pharma GmbH & Co KG Birkendorfer Straße 65 88397 Biberach Germany; ^3^ NanoTemper Technologies GmbH Floessergasse 4 81369 Muenchen Germany; ^4^ Leica Microsystems AG Max Schmidheiny-Strasse 201 9435 Heerbrugg Switzerland

**Keywords:** β-catenin, fragment-based screening, microscale thermophoresis, WaterLOGSY, PROTAC

## Abstract

Aberrant WNT pathway activation, leading to nuclear accumulation of β‐catenin, is a key oncogenic driver event. Mutations in the tumor suppressor gene APC lead to impaired proteasomal degradation of β‐catenin and subsequent nuclear translocation. Restoring cellular degradation of β‐catenin represents a potential therapeutic strategy. Here, we report the fragment‐based discovery of a small molecule binder to β‐catenin, including the structural elucidation of the binding mode by X‐ray crystallography. The difficulty in drugging β‐catenin was confirmed as the primary screening campaigns identified only few and very weak hits. Iterative virtual and NMR screening techniques were required to discover a compound with sufficient potency to be able to obtain an X‐ray co‐crystal structure. The binding site is located between armadillo repeats two and three, adjacent to the BCL9 and TCF4 binding sites. Genetic studies show that it is unlikely to be useful for the development of protein–protein interaction inhibitors but structural information and established assays provide a solid basis for a prospective optimization towards β‐catenin proteolysis targeting chimeras (PROTACs) as alternative modality.

## Graphical abstract

**Targeting the armadillo repeat**: We report the fragment‐based discovery of a small‐molecule binder to the key cancer target β‐catenin. Iterative virtual screening and ligand‐based NMR techniques enabled hit optimization in absence of structural information. The co‐crystal structure of a derivative in complex with a short β‐catenin variant revealed a novel small‐molecule binding site between armadillo repeats two and three.

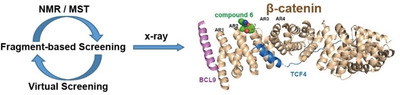

The WNT signal transduction cascade is a key regulatory pathway during embryonic development and adult tissue homeostasis. In the canonical WNT pathway, the armadillo repeat protein β‐catenin serves as the central signaling molecule, linking WNT ligand mediated pathway activation to target gene induction in the nucleus.^[1]^ In the absence of WNT ligands, signal transduction is tightly controlled by rapid proteasomal turnover of β‐catenin, mediated by a destruction complex consisting of the scaffolding proteins APC and Axin and two serine‐threonine kinases (CK1α and GSK3α/β).[^2^, ^3^] In cancer, aberrant WNT pathway activation is frequently observed. In particular, loss‐of‐function mutations in the tumor suppressor gene APC are present in approximately 80 % of colorectal cancers, resulting in impaired recruitment of β‐catenin to the destruction complex and WNT ligand‐independent stabilization of β‐catenin.[^1^, ^4^] Upon nuclear translocation, β‐catenin interacts with transcription factors of the TCF/LEF family to induce WNT target gene expression via recruitment of transcriptional co‐activators, such as CBP and BCL9.[^5^, ^6^, ^7^, ^8^]

Genetic inhibition of β‐catenin impairs growth of WNT pathway driven *in* *vitro* and *in* *vivo* tumor models,[^9^, ^10^, ^11^, ^12^] highlighting pharmacological inhibition of β‐catenin as a promising opportunity for targeted therapy of WNT pathway driven cancers. However, despite decades of efforts attempting to modulate the function of β‐catenin, direct targeting of the protein has remained an unreachable goal for conventional small molecule discovery. Phenotypic screening approaches led to the identification of a number of indirect modulators of β‐catenin.^[13]^ The most progressed small molecules bind to the *N*‐terminal domain of the coactivator, CBP, thereby antagonizing its interaction with β‐catenin.^[14]^ One of these, PRI‐724, has reached phase II of clinical development, outside of the cancer setting, for Hepatitis B and C derived liver cirrhosis.[^15^, ^16^] In addition to classical screening approaches, virtually designed peptide derived macrocycles were reported, inhibiting the interaction of the N‐terminal region of TCF and β‐Catenin, resulting in downregulation of target genes.^[17]^ A validated, chemistry‐derived, binder to β‐catenin, ideally with molecular interactions elucidated by X‐ray crystallography would offer options to target β‐catenin directly. Until recently, the only molecules fulfilling these criteria for β‐catenin were hydrocarbon‐stapled peptides, based on its natural binding partner Axin, bringing the burden of high molecular weight.[^18^, ^19^] Encouraged by the report of a short β‐catenin protein variant (β‐catenin^141–305^) comprising only the first four armadillo repeats (Figure 1a) and amenable for protein NMR applications,^[20]^ we set out to apply fragment‐based screening methods to the identify novel small molecule binding sites on β‐catenin.

**Figure 1:**
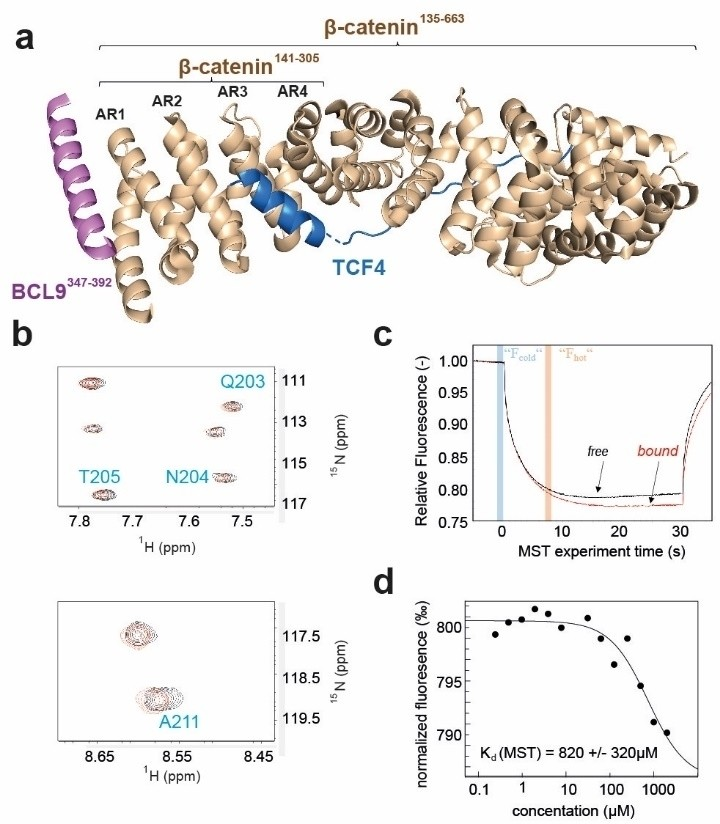
(**a**) Representation of the protein constructs used in this study based on the crystal structure of the β‐catenin‐BCL9‐Tcf4 complex (PDB ID: 2GL7). Proteins are shown as cartoon (BCL9 in violet, TCF4 in marine, and β‐catenin in wheat). The first four armadillo repeats (AR) comprised in β‐catenin^141–305^ are labeled respectively. (**b**) Sections of superimposed 2D ^15^N TROSY spectra of β‐catenin^141–305^ in the absence (black) or presence of 500 μM compound **1** (red) with cross peaks exhibiting observable minor chemical shift perturbation (full spectrum shown in Supporting Information Figure 2). Assignments were transferred from de la Roche et al.^[18]^ (**c**) MST screening data of 50 nM fluorescently labeled β‐catenin^141–305^ in the presence of 1 % DMSO as negative control (black) or 500 μM compound **2** (red). Time points of fluorescence readings for Δ*F*_nom_ calculation are highlighted in blue and orange, respectively. (**d**) MST *K*_d_ determination of compound **2**; 50 nM labeled β‐catenin^141–305^ with increasing concentrations of **2**.

We applied two parallel fragment‐based screening approaches using recombinant β‐catenin^141–305^ and a proprietary fragment library composed of 1899 compounds, respectively. In one cascade, we used saturation transfer difference nuclear magnetic resonance (STD‐NMR) spectroscopy and consecutive confirmation in two‐dimensional ^1^H/^15^N‐transverse relaxation optimized spectroscopy (TROSY) NMR experiments. Initially, the fragments were screened in mixtures of four compounds per well with STD NMR, and 145 hits were identified (Supporting Information Figure 1). From the primary hits 34 were confirmed in 2D ^15^N TROSY NMR experiments, where only minor chemical shift perturbations (CSPs) could be detected. To evaluate potential binding to the BCL9 site we mapped the epitope of the BCL9^347−392^ peptide by transferring the assignment from de la Roche et al.^[20]^ (Supporting Information Figure 2). However, as shown for compound **1** (Figure 1b, Supporting Information Figure 3) the observed CSPs are non‐overlapping to the BCL9 site.

**Figure 2:**
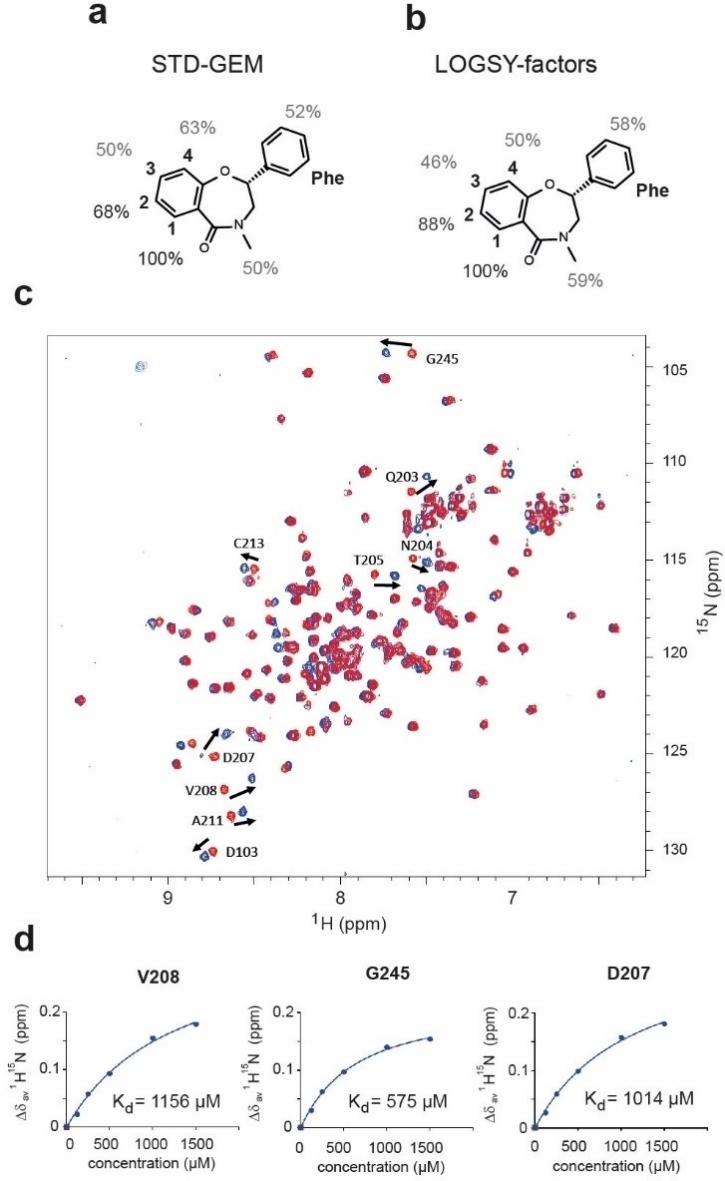
(**a**) STD‐GEMs and (**b**) LOGSY‐factors for compound 4 (**c**) Superposition of two‐dimensional ^15^N NMR spectra of 80 μM uniformly ^15^N labeled β‐catenin^141–305^ in absence (red) or presence (blue) of 500 μM compound 6. (**d**) Titration curves of selected cross peaks resulting in an averaged *K*_d_ of 915±303 μM (*x*‐axis: (μM), *y*‐axis Δ*δ*_av_(^1^H,^15^N) (ppm)).

**Figure 3:**
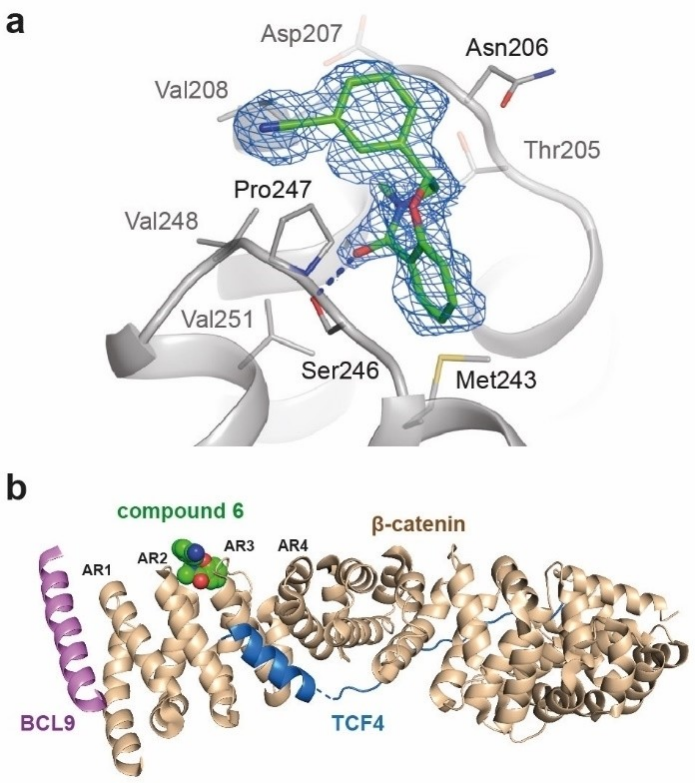
(**a**) Binding mode of compound **6** observed in the crystal structure in complex with β‐catenin^141–305^. Compound **6** is shown as stick model, color coded by atom type with carbon shown in green. The refined 2Fo‐Fc electron density shown in blue is contoured at 0.8 σ (**b**) Superposition of compound 6 from the crystal structure (protein not shown) with the crystal structure of the β‐catenin‐BCL9‐Tcf4 complex (PDB ID: 2GL7) (Cα RMSD=0.46 Å). Proteins are shown as cartoon (BCL9 in violet, TCF4 in marine, and β‐catenin in wheat), compound **6** is shown as spheres, color coded by atom type with carbon shown in green. The first four armadillo repeats (AR) comprised in β‐catenin^141–305^ are labeled respectively.

As a second fragment finding approach, we applied Microscale Thermophoresis (MST). Initially, the screening assay was validated by a titration with recombinant BCL9^347−392^ revealing a *K*_d_ of 360 nM (Supporting Information Figure 4) which is in good agreement with the previously determined binding affinity using ITC (*K*_d_=540 nM).^[21]^ The outcome of the MST detected FBS was comparable to the STD‐NMR screen, yielding 134 primary hits. Compound **2** was identified as a binder to β‐catenin^141–305^ with a significant binding signal of ΔΔ*F*_nom_ of 7.5‰ compared to standard deviation with DMSO of 1.8‰ (Figure 1c) Subsequent titration experiments confirmed 16 hits as dose dependent binders with affinities in the high three digit micromolar to millimolar range, as shown for compound **2** (MST; *K*_d_ =820±320 μM, Table 1, Figure 1d). Despite extensive co‐crystallization experiments with hits from both screening cascades, no co‐crystal structure with β‐catenin^141–305^ could be obtained.

**Table 1:** Molecular structure, solubility and affinity data for compounds **1**–**7**.

Compound		Solub. pH 7.5 [μM]	HSQC (MST*) *K*_d_ [μM]^[a]^	SPR *K*_d_ [μM]^[b]^
**1**	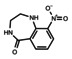	>500	>10,000	n.d.
**2**	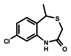	>500	820±320*	n.d.
**3**	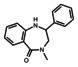	1000	1100±255	n.d.
**4**	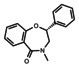	>2000	2260±1260	>10,000
**5**	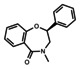	>2000	>10,000	>10,000
**6**	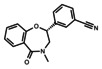	>2000	915±303	1390±46
**7**	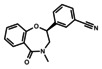	>2000	>10,000	>10,000

[a] MST and NMR *K*_d_ values originate from single titrations, mean *K*_d_ values obtained from curves of selected cross peaks±standard deviations. [b] SPR *K*_d_ values reported as mean of three independent experiments±standard deviations.

In order to progress hit finding in the absence of structural information, an automated workflow for ligand‐based virtual screening was employed. All identified hits were used as query structures in multiple parallel similarity searches against the Boehringer Ingelheim (BI) corporate compound collection, utilizing a panel of different similarity search methods and metrics succeeded by data fusion,^[22]^ this serves as BI's “SAR by catalog” framework.^[23]^ In total, 200 virtual screening hits were selected and analyzed in ^15^N TROSY NMR detected titration experiments. The racemic mixture of compound **3** showed the strongest CSPs and was titrated to yield an average *K*_d_ of 1100 μM, which had to be extrapolated because the solubility of the compound was limited at 1000 μM (Table 1, Supporting Information Figure 5). Compound **3** was therefore used as novel query structure in a follow‐up search using the same approach as described above. The racemic mixture of compound **4** and **5** was identified, maintaining the CSP pattern. For further analysis the enantiomers where separated and subjected to ^15^N NMR detected titrations, yielding the eutomer compound **4** (NMR; *K*_d_ =2260±1260 μM; Table 1, Supporting Information Figure 6) and the distomer compound **5** (NMR; *K*_d_ >10 mM, Table 1) and their solubility was determined to be >2000 μM, allowing for a higher titration.

Given the lack of detailed structural information at this stage of the project, conventionally provided by X‐ray crystallography, we performed ligand‐based NMR techniques‐GEM (group epitope mapping) by STD^[24]^ and WaterLOGSY‐titration experiments[^25^, ^26^, ^27^]‐to evaluate the binding mode of compound **4**. STD and WaterLOGSY experiments provide information about protein‐buriedness and solvent‐exposure of ligand protons in the bound state. Figure 2 shows a comparison between STD GEMs (Figure 2a), extracted from STD build‐up curves, and LOGSY‐factors (Figure 2b, Supporting Information Figure 7) extracted from titration experiments for compound **4**.

The larger β‐catenin^135–663^ construct was used, as STD‐ and WaterLOGSY‐experiments are more efficient with larger proteins and the observed ligand signals are not affected by underlying protein signals, usually observed with small protein constructs. There is a good consensus between the two methods, regarding the proton that is most deeply buried and has the closest contact to the protein, as indicated by the highest percentages. Both the STD GEMs and the LOGSY‐factors indicated that the phenyl ring is only partly buried and there might be an opportunity to grow into unoccupied binding site space. Hence a small series of phenyl derivatives were synthesized. The racemic mixture of compound **6** and **7** showed an improved CSP pattern and was subsequently subjected to chiral separation. The consecutive NMR *K*_d_ titration revealed that eutomer **6** exhibits improved affinity (NMR; *K*_d_=915±303 μM, Table 1, Figure 2c and 2 d) whereas distomer **7** did not show any CSPs at a concentration of 500 μM (Supporting Information Figure 8).

Compound **6** finally enabled the co‐crystal structure determination in complex with β‐catenin^141–305^ at a resolution of 1.98 Å (Figure 3a, Supporting Information Figure 9). The ligand was found to bind to a solvent‐exposed pocket in a loop region between armadillo repeats 2 (residues 205–210) and 3 (residues 243–251) (Figure 3b).^[28]^

The ligand's distinct three‐dimensional shape complements the pocket while orienting the N‐methyl lactam functionality towards the receptor. Thereby, the carbonyl moiety of compound **6** forms a hydrogen bond to the hydroxyl group of Ser246. Additionally, the fused aryl ring forms CH‐π interactions with the backbone CH of Ser246 while orienting an ortho aryl CH towards the sulfur lone pairs of Met243. The meta‐cyano‐phenyl substituent orients orthogonal to the ligand's bicyclic core, binding to a hydrophobic area involving Val208 and Val248. The cyano substituent is solvent‐exposed, potentially acidifying the para CH via electron withdrawal and thus strengthening its interactions with the backbone carbonyl of Asn206. Based on the strong validation of the eutomer(**6**)‐distomer(**7**) pair, we attempted SPR analysis, which could serve as a high‐throughput assay for consecutive optimization cycles. The assay was validated using BCL9^347−392^ as a positive control (SPR; *K*_d_=225 nM±3.5, Supporting Information Figure 10) In comparison, the *K*_d_ for eutomer **6** is weak (SPR: **6**; *K*_d_=1390±46 μM) and data could only be acquired up to a concentration of 1 mM and the fit curve is extrapolated (Supporting Information Figure 11a). The sensorgrams show contribution from non‐specific binding and an over‐stoichiometric binding response is observed. Interestingly, distomer **7** shows a comparable non‐specific binding effect in the absence of any saturable behavior (SPR: **7**; *K*_d_>10 mM, Supporting Information Figure 11b).

The overlay of the co‐crystal structure of compound **6** with the crystal structure of a β‐catenin‐BCL9‐Tcf4 complex^[21]^ (PDB ID: 2GL7) suggests that binding of compound **6** does not interfere with binding of the selected transcription factors TCF4 and BCL9 (Figure 3b). To assess the potential functional relevance of the compound binding site, TOPFlash reporter gene assays were performed (Supporting Information Figure 12a). The TOPFlash reporter plasmid comprises a TCF‐driven firefly luciferase, allowing for luminescence based readout of β‐catenin/TCF transcriptional activity. Transfection of β‐catenin (control) or β‐catenin variants harboring substitutions in the compound binding site (A211F, S246V, A211F_S256V) resulted in comparable protein expression levels in HEK293 cells (Supporting Information Figure 12a). In the reporter gene assay, β‐catenin variants showed similar transcriptional activity, in comparison to the β‐catenin control construct (Supporting Information Figure 12a). In particular, comparable reporter gene activation to the β‐catenin control was observed for the A211F_S256V double mutant variant. In β‐catenin co‐immunoprecipitation studies with TCF4 and BCL9, we further tested whether the compound binding site is relevant with respect to PPI interactions with co‐activators. To this end, β‐catenin (control) or compound binding site variants (A211E, A211F_S246V, A211E_S246E, A211I_S246F) were co‐expressed in HEK293 and co‐immunoprecipitation of TCF4 and BCL9 was monitored upon pull‐down of β‐catenin using immunoblotting (Supporting Information Figure 12b). In comparison to β‐catenin (control), all variants showed similar co‐immunoprecipitation of TCF4 and BCL9, indicating that interactions of β‐catenin with TCF4 or BLC9 were not affected by introduction of amino acid substitutions in the compound binding site. In line with previous structural and functional studies,^[28]^ the results of the reporter gene and co‐immunoprecipitation studies imply that addressing this binding site might not affect β‐catenin function.

Despite significant progress made with stapled peptide based approaches, β‐catenin is still deemed by most as “undruggable”. Here, we describe the identification of a fully validated small molecule binder to a newly discovered binding site on β‐catenin. To our knowledge, it is the first time that the key cancer target β‐catenin was successfully screened with fragment‐based methods, leading to a co‐crystal structure with biophysically determined *K*_d_. The initial screening efforts based on NMR and MST approaches only delivered weak hits, which hindered the structure determination by X‐ray crystallography. We therefore applied alternative strategies to bootstrap the classical fragment optimization and progress hits in the absence of structural information.^[29]^ Both pursued approaches, iterative ligand‐based virtual screening and ligand‐based NMR techniques, GEM by STD and WaterLOGSY‐titrations, are generally applicable to FBS projects. Particularly, the latter ones proved to be valuable tools as they focused the area of chemical space explored during the analoging process. Compound 6 binds to β‐catenin with an affinity of 915 μM and the co‐crystal structure provides a path for further optimization. The novel, unexplored binding site was characterized with respect to potential interference with binding of the transcription factors TCF4 and BCL9, using co‐immunoprecipitation studies. The experiments suggest that the identified binding site is non‐functional, and thereby is unlikely to be applicable for the development of protein‐protein interaction inhibitors of TCF4 for example, which binds tightly to β‐catenin (∼20 nM).^[30]^ As oncogenic mutations stabilize β‐catenin and thereby lead to elevated protein levels, restoring cellular degradation is a potential therapeutic strategy. As oncogenic mutations stabilize β‐catenin and thereby lead to elevated protein levels, restoring cellular degradation is a potential therapeutic strategy. This concept lead to the discovery of molecular glue‐like small molecules enhancing the interaction of mutant β‐catenin and the natural E3 ligase β‐TrCP in engineered mutant β‐catenin cells.[31] Proteolysis targeting chimeras (PROTACs) are a promising strategy to degrade and target β‐catenin. In‐vivo proof of concept for this approach was recently achieved using stapled peptide based PROTACs recruiting the E3 ligase VHL.[32] The discovered eutomer‐distomer pair provides a high level of confidence that can support ligand optimization and consecutive enablement of β‐catenin targeting PROTACs.

## Data availability

The authors declare that the data supporting the findings of this study are available within the publication and its Supporting Information. The coordinates of the crystal structure of β‐catenin^141–305^ in complex with compound **6** have been deposited in the RCSB Protein Data Bank (PDB; http://www.rcsb.org) with the accession number 7AFW.

## Competing interests

D.K., M.M., S.K.Z., M.Z., S.W., A.B., J.Br., T.C., G.D., M.D., S.D., J.E.F., L.G., W.H., C.K., R.K., F.M., T.P., K.R., M.S., P.W., B.W., M.P., D.B.M., and J.Bö. are employees of Boehringer Ingelheim.

## Supporting information

As a service to our authors and readers, this journal provides supporting information supplied by the authors. Such materials are peer reviewed and may be re‐organized for online delivery, but are not copy‐edited or typeset. Technical support issues arising from supporting information (other than missing files) should be addressed to the authors.

Supplementary
