# Supplementary material for: Getting a Grip on the Undrugged: Targeting β‐Catenin with Fragment‐Based Methods
Source: ChemMedChem. 2021 Jan 14;16(9):1420–4. doi: 10.1002/cmdc.202000839 (PMC8247886; doi:10.1002/cmdc.202000839)
Supplement: Supplementary file 1 — Supplementary [file CMDC-16-1420-s001.pdf]

ChemMedChem

Supporting Information

Getting a Grip on the Undrugged: Targeting β-Catenin with Fragment-Based Methods

Dirk Kessler[+],* Moriz Mayer[+], Stephan K. Zahn[+], Markus Zeeb, Simon Wöhrle, Andreas Bergner, Jens Bruchhaus, Tuncay Ciftci, Georg Dahmann, Maike Dettling, Sandra Döbel, Julian E. Fuchs, Leonhard Geist, Wolfgang Hela, Christiane Kofink, Roland Kousek, Franziska Moser, Teresa Puchner, Klaus Rumpel, Maximilian Scharnweber, Patrick Werni, Bernhard Wolkerstorfer, Dennis Breitsprecher, Philipp Baaske, Mark Pearson, Darryl B. McConnell, and Jark Böttcher*

Table 1. Data collection and refinement statistics

**Compound**	**6**
**Data collection**	
**Resolution Limit defined by**	STARANISO
Space group	$P\ 3\ 2_1$
Cell dimensions	
a, *b*, *c* (Å)	53.45, 53.45, 87.55
α, β, γ (°)	90.00, 90.00, 100.00
Resolution (Å)	1.81 (2.00)*
R_{merge}	7.5
$I / \sigma I$	24.4
Completeness (%)	88.4 (95.6)
Redundancy	8.3
**Refinement**	
Resolution (Å)	1.81
No. reflections	11045
R_{work} / R_{free}	22.7/28.1
No. atoms	
Protein	1224
Ligand/ion	21
Water	69
B-factors	
Protein	18.93
Ligand/ion	22.22
Water	22.22
R.m.s. deviations	
Bond lengths (Å)	0.009
Bond angles (°)	0.97

[a] approximate effective resolution in parentheses
[b] resulting completeness after STARANISO for spherical / elliptical shells

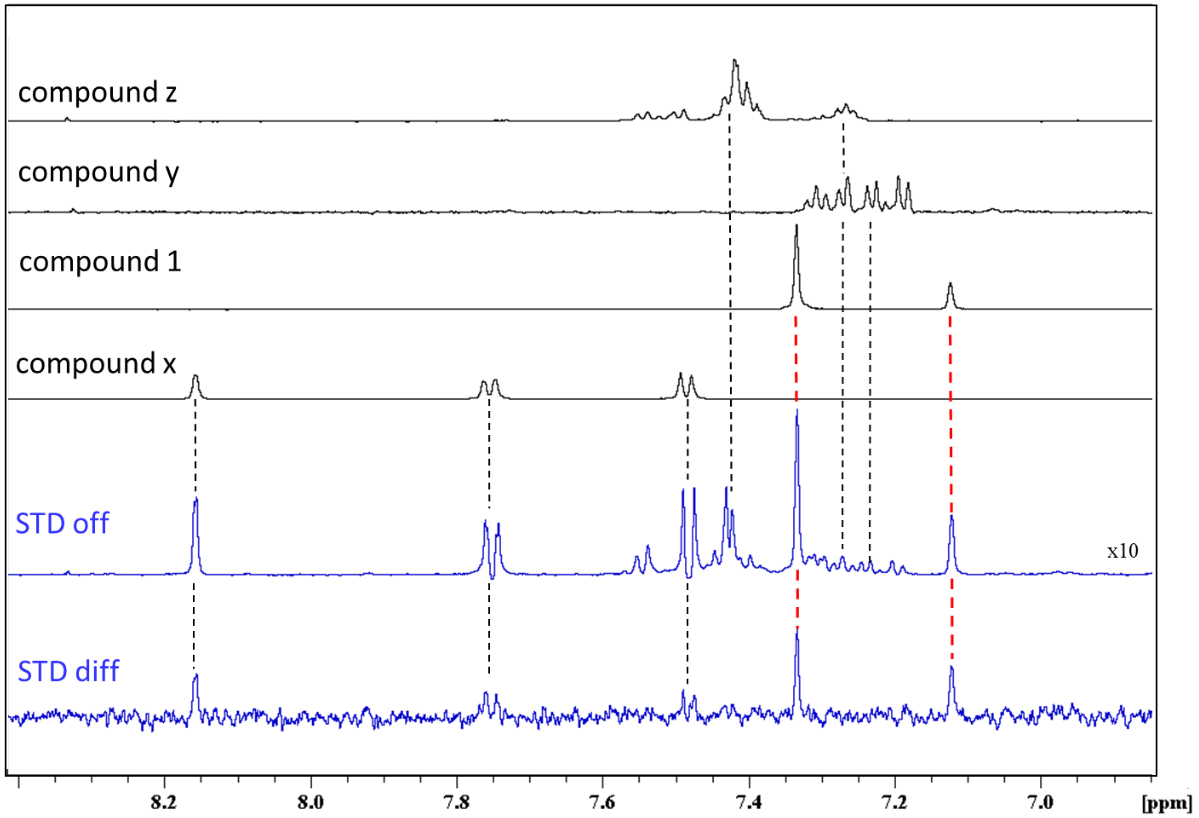

Supplementary Figure 1: Aromatic region of the ^{1}H NMR spectrum. From top to bottom: in black pre-recorded reference spectra of compound **1** and compounds x, y, z (not disclosed) present in the fragment mixture; in blue STD off-resonance and STD difference spectra are shown. Assignment of resonances in the mixture to individual compounds highlighted with dotted lines. Compounds x and **1** are unambiguously identified as binders to β-catenin$^{141\text{-}305}$. Red dotted lines indicate the compound **1**, which is described in the text.

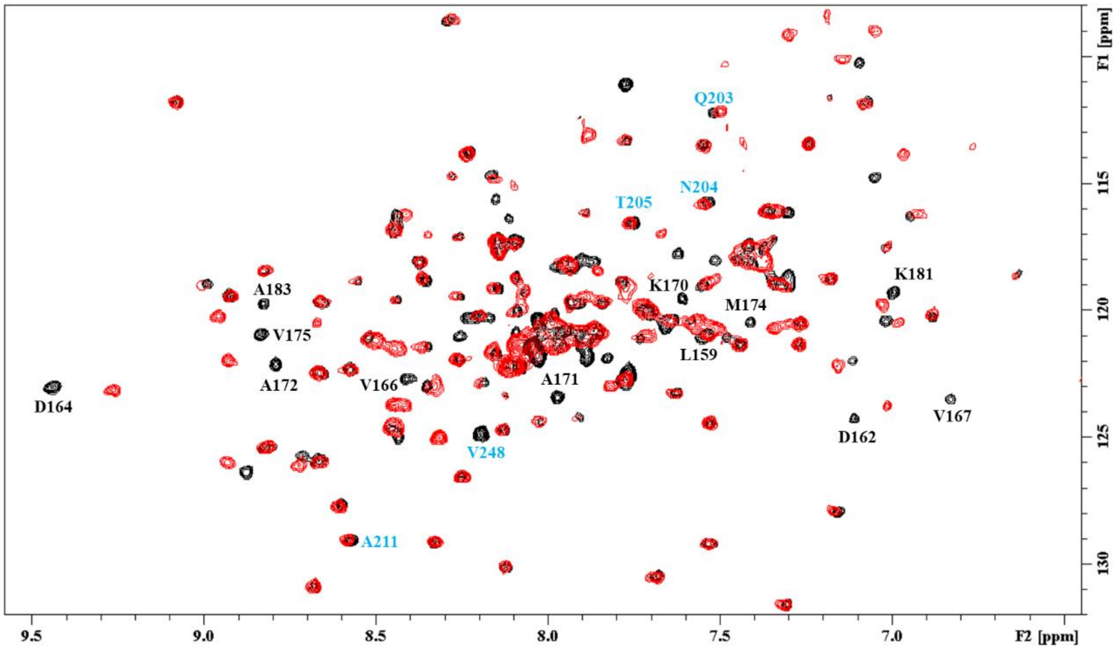

Supplementary Figure 2: Superposition of two-dimensional ^{15}N TROSY NMR spectra of 100 µM uniformly ^{15}N labeled β-catenin$^{141\text{-}305}$ in the absence (black) and presence (red) of 100 µM BCL9$^{347\text{-}392}$. Cross peaks of main interacting residues with largest CSPs are highlighted in black. Cross peak assignments were adapted from de la Roche et al[1]. Cross peaks marked in blue show minor chemical shift perturbation with Compound **1**.

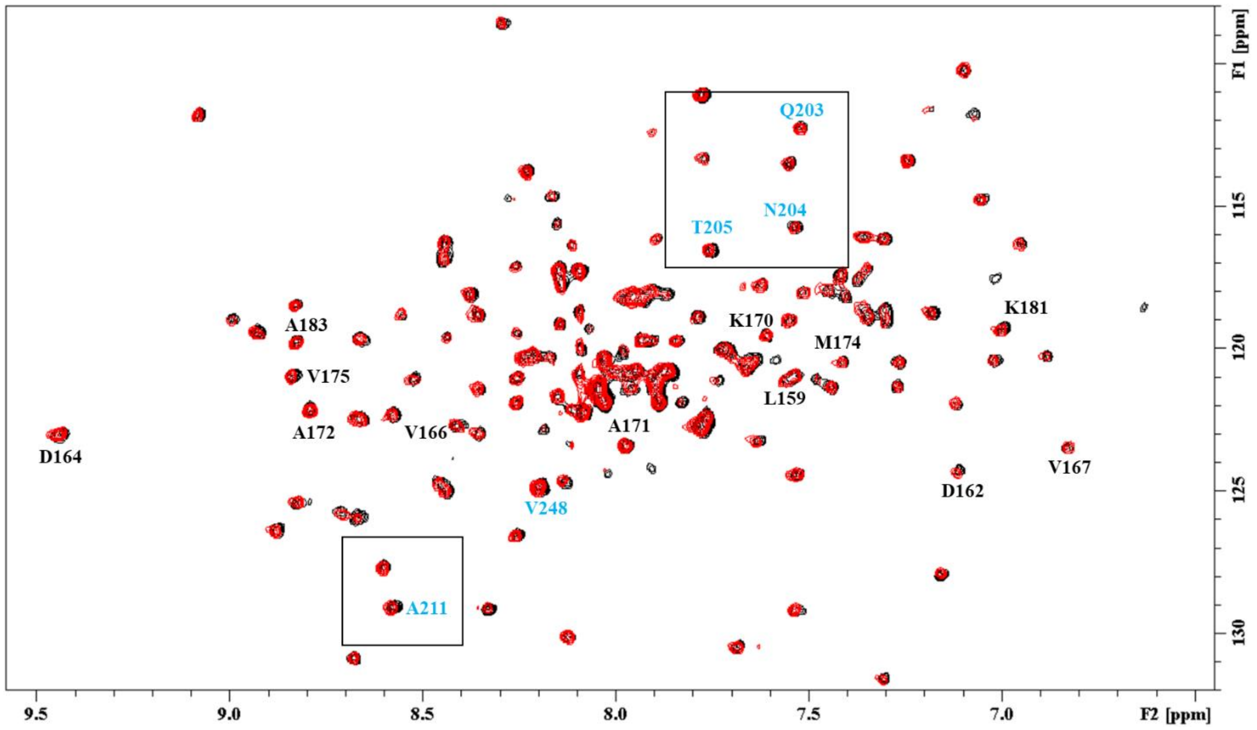

Supplementary Figure 3: Superposition of two-dimensional ^{15}N TROSY NMR spectra of 100 μM uniformly ^{15}N labeled β-catenin$^{141-305}$ in the absence (black) and presence (red) of 500 μM compound **1**. Cross peaks marked in blue show minor chemical shift perturbation with Compound **1**. Areas of selected cross peaks shown in Fig 1b. are highlighted with black squares. Marked cross peaks in black identify the residues of β-catenin$^{141-305}$ experiencing the largest CSPs upon complex formation with BCL9$^{347-392}$. Cross peak assignments were adapted from de la Roche et al[1].

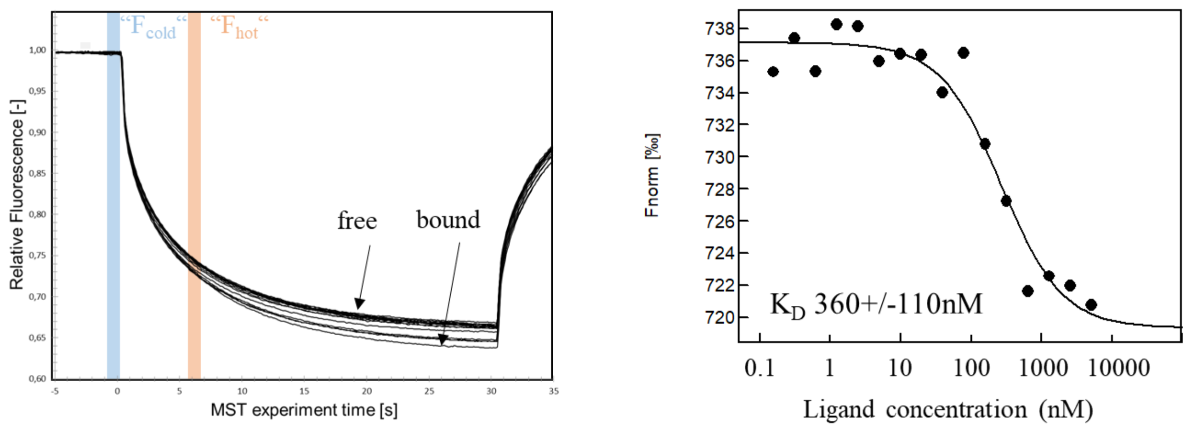

Supplementary Figure 4: (**a**) MST traces of 50 nM fluorescently labeled β-catenin$^{141-305}$ in the presence of increasing concentrations of BCL9$^{347-392}$. (b) MST K_d determination of β-catenin$^{141-305}$ with BCL9$^{347-392}$ in PBS pH 6.8 supplemented with 100 mM $NaNO_3$, 0.5 mM TCEP and 0.05% Tween-20 at 25 °C.

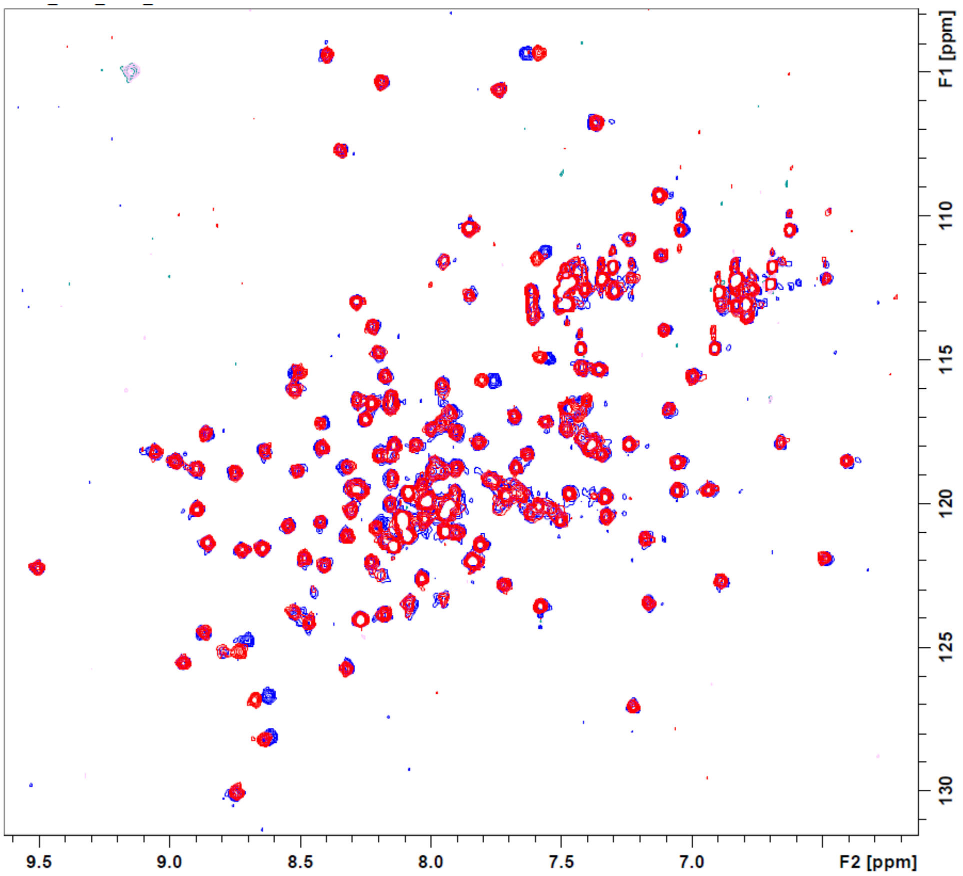

Supplementary Figure 5: Superposition of two-dimensional ^{15}N HMQC NMR spectra of 70 μM uniformly ^{15}N labeled β-catenin$^{141-305}$ in the absence (red) and presence (blue) of 500 μM compound **3**.

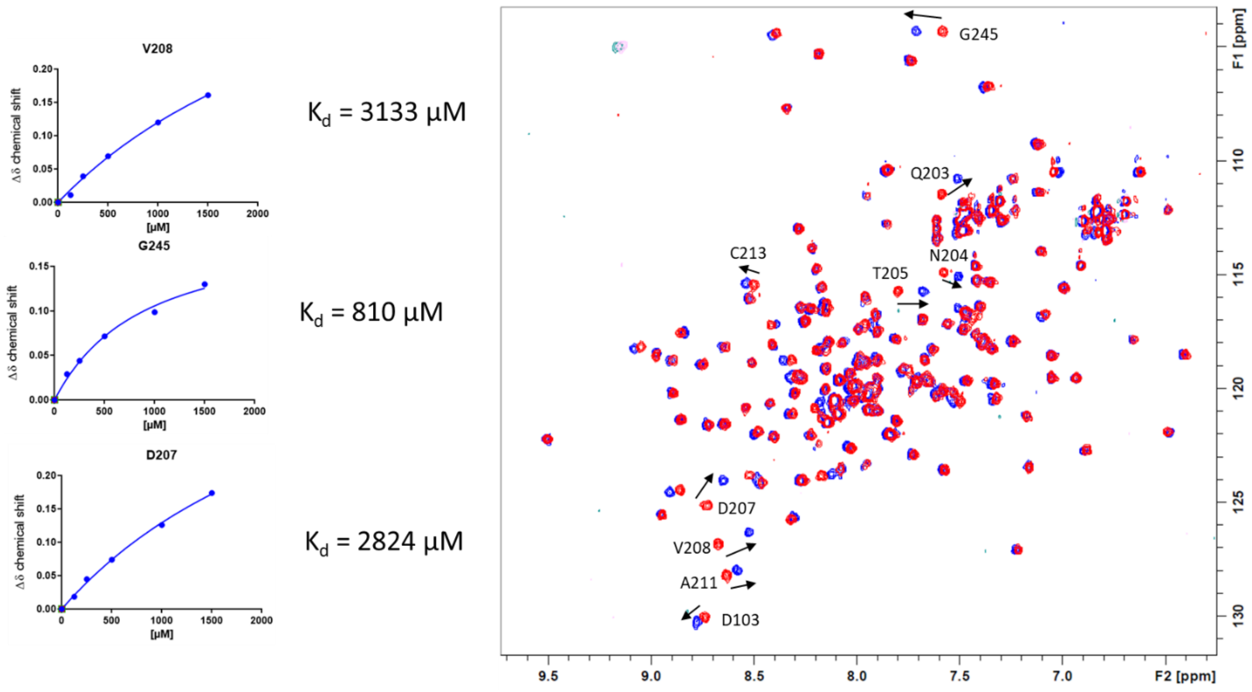

Supplementary Figure 6: Superposition of two-dimensional ^{15}N HMQC NMR spectra of 70 μM uniformly ^{15}N labeled β-catenin$^{141\text{-}305}$ in the absence (red) and presence (blue) of 500 μM compound **4**.

Titration curves of selected cross peaks yielding resulting in an averaged K_d of 2260 +/-1260 μM (x-axis: (μM), y-axis $\Delta\delta av(^1H,^{15}N)$ (ppm)).

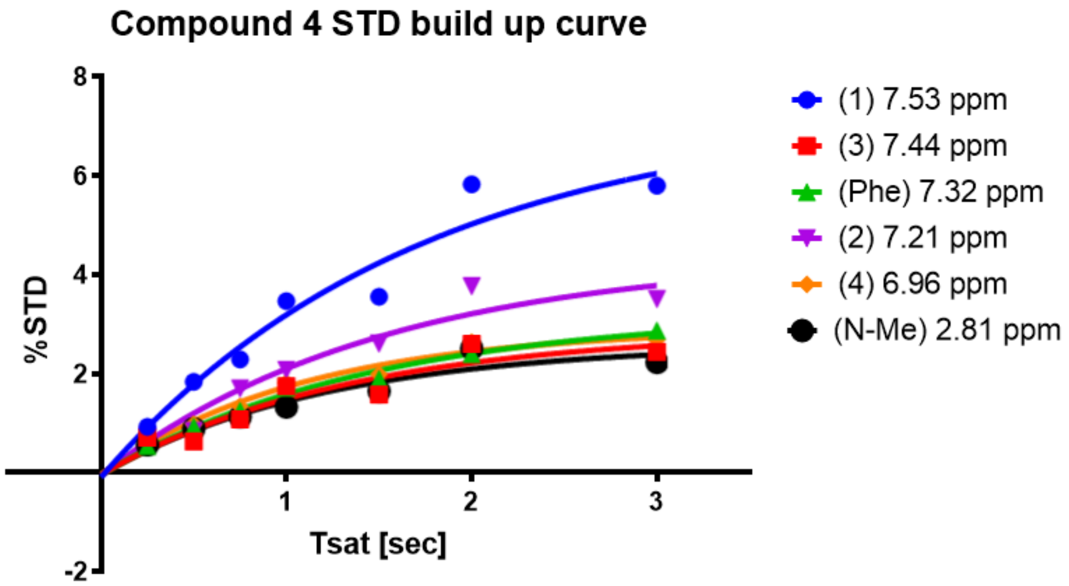

Supplementary Figure 7: STD build up data of compound **4** to obtain initial rate constants of the STD that are independent of the T_1 times of the individual protons[2]. Data was fit with Graphpad Prism 8

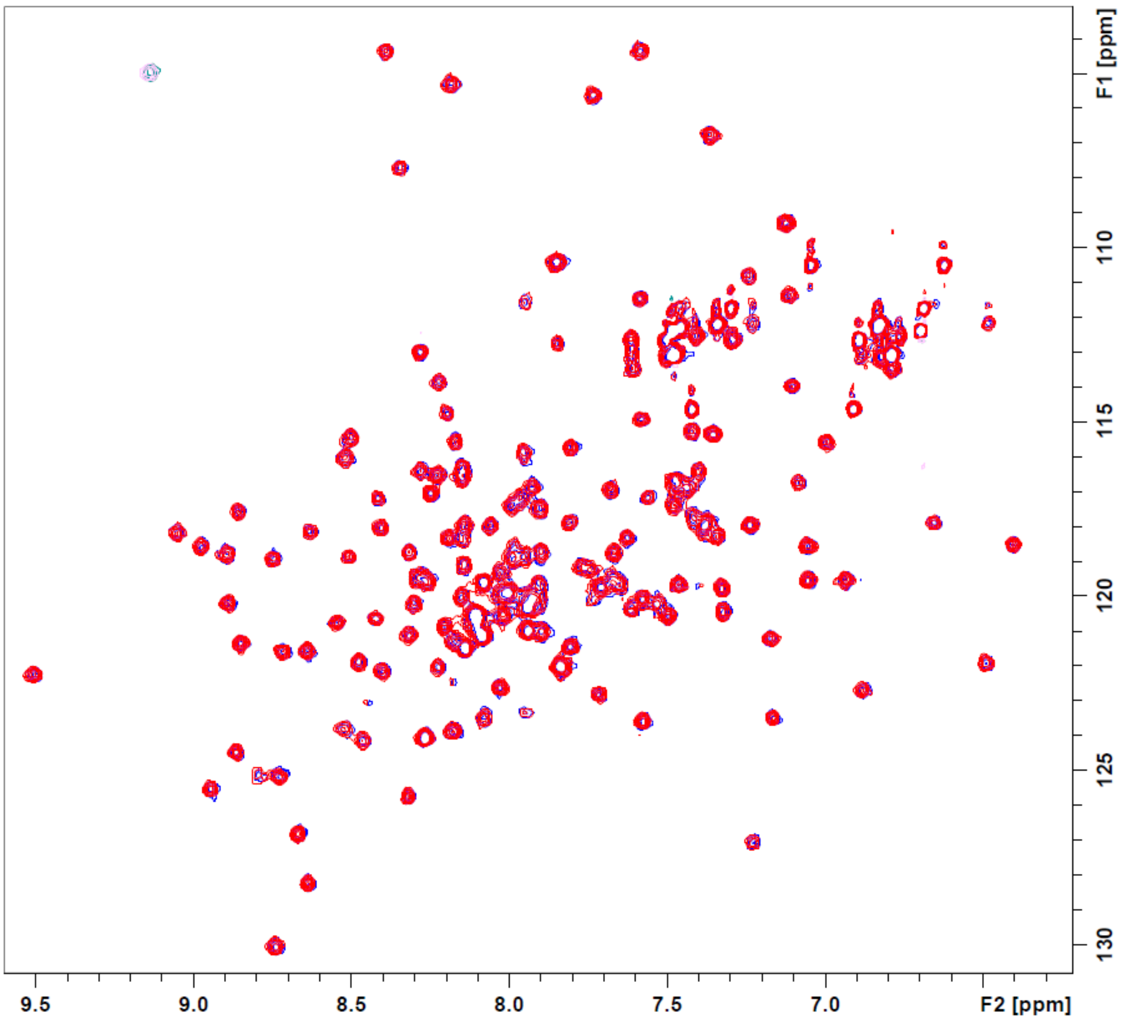

Supplementary Figure 8: Superposition of two-dimensional ^{15}N HMQC NMR spectra of 70 µM uniformly ^{15}N labeled β-catenin$^{141\text{-}305}$ in the absence (red) and presence (blue) of 500 µM distomer **7**.

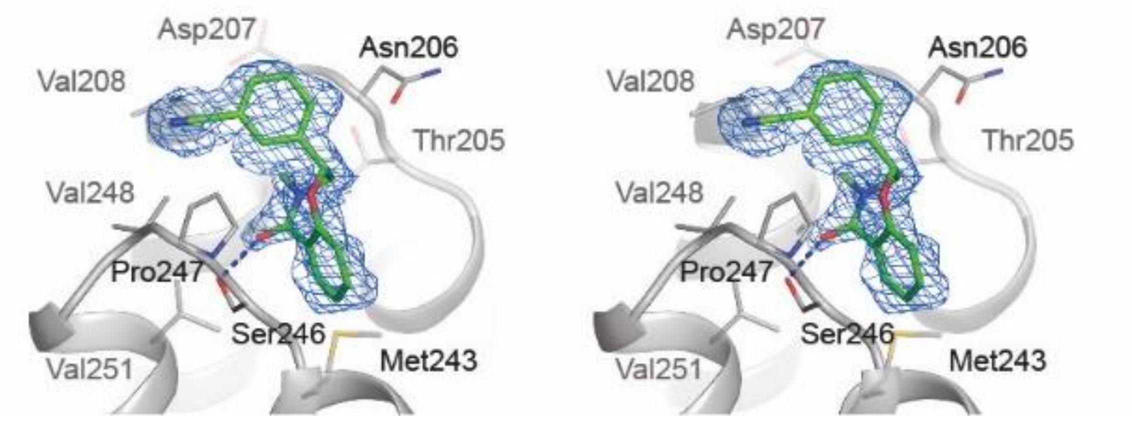

Supplementary Figure 9: Stereo image of compound **6** (color coded by atom type) bound to β-catenin (wall-eye stereo). The refined $2F_o$-F_c electron density is contoured at 0.8 σ.

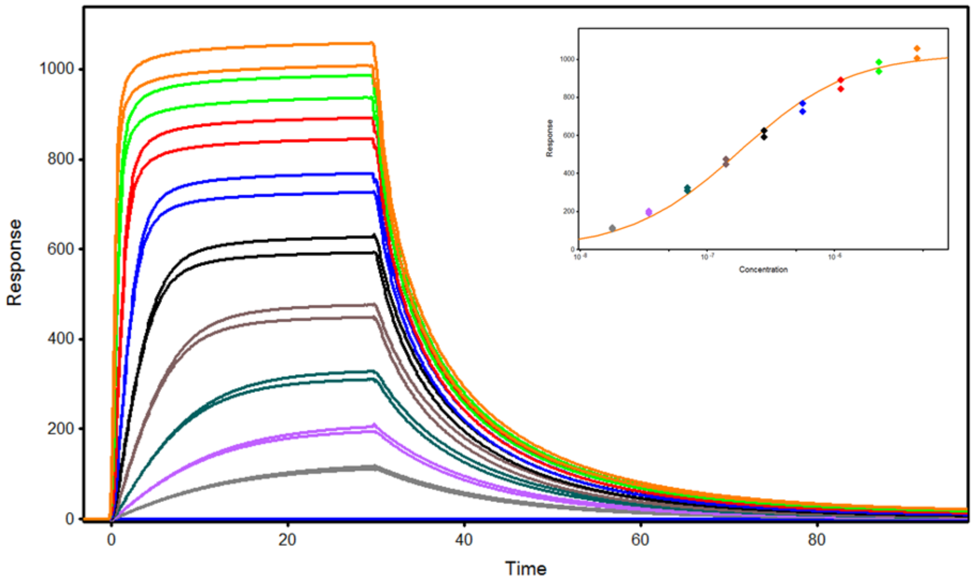

Supplementary Figure 10: SPR data for binding of BCL9$^{347-392}$ to β-catenin$^{141-305}$ (n=3). The inset shows the fit of the equilibrium responses using a 1:1 binding model, yielding a K_d of 225 nM +/- 3.5.

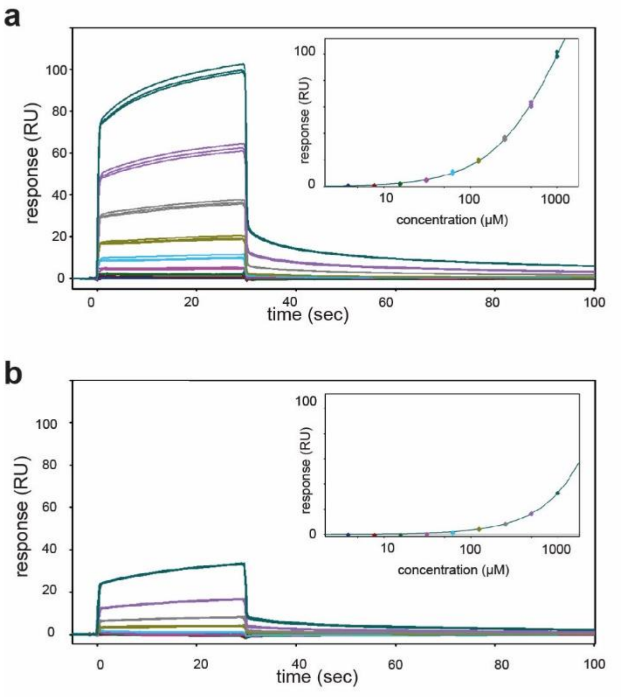

Supplementary Figure 11: SPR data for binding of compound **6 (a)** and **7 (b)** to β-catenin$^{141\text{-}305}$ (n=3). The inset shows the fit of the equilibrium responses using a 1:1 binding model (SPR: **6**; K_d = 1390 +/-46 μM; **7**: K_d > 10 mM).

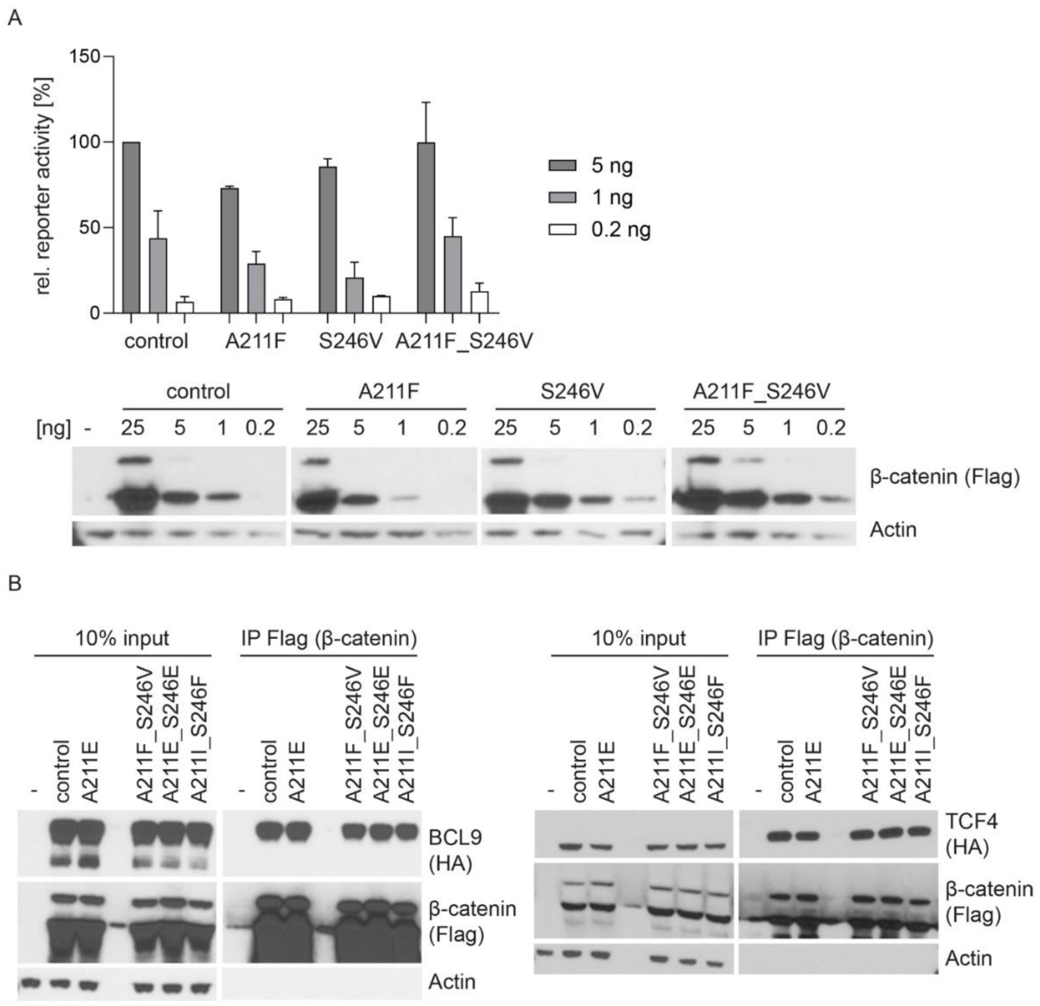

Supplementary Figure 12: Assessment of the functional relevance of the compound binding site using β-catenin variants harboring mutations in the compound binding pocket. (A) TOPFlash reporter gene assays in HEK293 cells upon transfection of active β-catenin (control) or binding pocket mutants (upper panel). Equal expression of control β-catenin or binding pocket mutants was monitored by immunoblotting (lower panel). (B) Co-immunoprecipitation studies in HEK293 cells transiently expressing active β-catenin (control) or binding pocket mutants. Immunoprecipitation of Flag-tagged β-catenin was performed using anti-Flag antibodies and co-precipitation of HA-tagged BCL9 (left panel) or HA-tagged TCF4 (right panel) was monitored using immunoblotting.

Online Methods

Protein purification

The construct for expression of β-catenin$^{141\text{-}305}$ (Amino acids 141-305 of uniprot ID: P35222) is based on the previously reported R4 construct[1]. The plasmid was used to transform *E. coli*, strain BL21 (DE3) cells (Invitrogen). For protein expression, an overnight culture in LB-medium supplemented ampicillin (100 µg/mL) at 37 °C was prepared and diluted the next day with fresh 2xYT-medium. At OD_{600} of 0.8 the culture was cooled down to 18 °C and after induction with 0.5 mM IPTG incubated for 20 h. Cell pellets obtained by centrifugation at 5000 rpm were stored at -20 °C. Cells were solubilized in buffer A (2X Dulbecco's PBS; 500 mM NaCl; 10 % Glycerol; 0.5 mM TCEP; cOmplete™ Protease Inhibitor Cocktail; pH 7.4) and disrupted by sonication (Sonopuls, Bandelin) on ice. After 30 min the sonicated lysate was clarified by centrifugation (50 min, 13500 rpm, 4 °C). The supernatant was mixed with 20 mL Glutathione Sepharose® 4B resin (GE Healthcare) slurry, washed in lysis buffer, and steered for 3 hours on ice. The beads were spun down, loaded into a XK16 column cartridge and washed with lysis buffer until baseline. The protein was eluted with buffer B (2X Dulbecco's PBS; 500 mM NaCl; 10 % Glycerol; 0.5 mM TCEP;; pH 7.4) + 25 mM L-Glutathione reduced. After cleavage with 2000 units Human Alpha Thrombin (Enzyme Research Laboratories) at room temperature overnight, the protein was collected in the flow-through fraction of a second GST run (GSTrap HP 5 mL column; GE Healthcare) using buffer B. The flow-through was concentrated by centrifugation using an Amicon© Ultra 15 mL Centrifugal Filter (Merck Millipore) and loaded onto a HiLoad Superdex S75 column (GE Healthcare) equilibrated with 50 mM Tris; 200 mM NaCl; 3 mM DTT; pH 8. Fractions were analyzed by SDS-PAGE and fractions containing the desired protein were pooled and concentrated with an Amicon© Ultra 15 mL Centrifugal Filter (Merck Millipore). For ^{15}N

labelling the protein was expressed in Overnight Express™ Autoinduction NMR Medium - ^{15}N - Novagen (Merck Millipore) according to the technical description and purified as described for the unlabeled protein. The protein of β-catenin$^{135\text{-}663}$ (Amino acids 135-663 of uniprot ID: P35222) and the β-catenin binding domain of human BCL9 (Amino acids 347–392 of uniprot ID: O00512, containing an additional n-terminal glycine) was purchased (Proteros Biostructures GmbH).

NMR spectroscopy

Ligand based 1D ^1H experiments were carried out on a Bruker Avance II 600 MHz spectrometer with a 5 mm cryo-QCI probe and z-gradients. An in house customized Tecan Freedom Evo liquid handler[3] was used to automatically prepare and transfer samples into 2.5 mm NMR tubes inserted in 96 in house modified Bruker MATCH systems to ensure identical incubation times. NMR samples were transported to the magnet with the Bruker Sample Rail system. Randomly combined mixtures of four fragments from 50 mM d6-DMSO stocks were tested at an individual concentration of 250 µM each in 25 mM sodium phosphate, 150 mM sodium chloride, 100 mM sodium nitrate, 0.5 mM TCEP pH 6.8 in D_2O at 25 °C. A protein concentration of 10 µM β-catenin$^{141\text{-}305}$ was used. For STD-NMR experiments a Gaussian pulse train with a duration of 3 s was employed to accomplish selective irradiation and a 30 ms spin-lock pulse was used to suppress residual protein signals. The on and off resonance spectra were recorded in an interleaved fashion to minimize subtraction artifacts. The irradiation frequency for the on- and off resonance experiment were -100 Hz and -40 kHz, respectively. After acquisition, the difference spectrum was calculated by subtracting the two individual spectra. Compound binding was observed by signals arising in the difference spectrum. These were readily assigned by comparing the signals with the respective pre-recorded reference spectra of the individual compounds present in the mixture. The quality control of the fragment library included purity, identity, and buffer solubility

measurements. To rule out false positives all library members were also tested for their tendency of self-association or micelle formation by STD experiments in the absence of protein.

For confirmation of hits obtained from STD-NMR by 2D ^{15}N TROSY NMR[4] a Bruker Avance III 600 MHz instrument with a 5 mm cryo-TCI probe and z-gradient was used. The experimental set up (automated sample preparation and transfer into the magnet by a Tecan Freedom Evo pipetting robot and a Bruker Sample Rail system, respectively) was identical. Each sample contained 25 uM ^{15}N labeled β-catenin$^{141\text{-}305}$ 25 mM sodium phosphate, 150 mM sodium chloride, 100 mM sodium nitrate, 0.5mM TCEP pH 6.8 in H2O at 25 °C and 8% (v/v) D_2O. The protein was incubated with 500 uM fragment in a 2.5 mm NMR tube at 25 °C and a d6DMSO concentration of 1%. Spectra were recorded with 48 transients and 64 data points in the indirect dimension. Manual comparison of the spectrum in the presence of the to be tested fragment with the DMSO reference spectrum reveal either line broadening or chemical shift perturbation of cross peaks. Both effects are indicative for compound binding. All processing and analysis was done with the Topspin 2.1 software (Bruker BioSpin). Further spectral comparisons were performed with the FELIX software package (Felix NMR Inc.) to combine manual with automated analysis[5].

1D, GEM by STD and WaterLOGSY experiments were recorded at 298 K on an Avance III 700MHz spectrometer equipped with a cryogenically cooled 5mm TCI probe. Spectra were processed and analyzed with Topspin 3.5 (Bruker BioSpin). The 1D spectra were recorded with a double WATERGATE[6] suppression element with the number of scans set to 32. The WaterLOGSY pulse sequence was used as described before[7]. Selective water inversion was achieved with a double excitation sculpting element employing water selective Gaussian refocusing pulses (inversion bandwidth of 0.5 ppm, pulse length of 7.5 ms). The number of scans was set to 2048. Sample concentrations were 0.5 mM ligand and 0.25–10 µM β-catenin$^{135\text{-}663}$ in

buffer (Tris 25 mM, pH 7.5, NaCl 150 mM) containing 2% DMSO-d6. A stock solution of β-catenin$^{135-663}$ (12.5 µM) was dialyzed before into Tris 25 mM pH 7.6 NaCl 150 mM and centrifuged at 4° C at 15000 rpm for 20 minutes. Intensity ratios I_{logsy}/I_{1D} are derived from scaling of the 1D intensity to the corresponding LOGSY intensity. STD GEMs were acquired with 1.5 µM β-catenin$^{135-663}$ and 500 µM compound **4**. Saturation time Tsat was set to 0.25; 0.5; 0.75; 1.0; 1.5; 2.0; and 3.0 seconds for the saturation time build up curves to determine the initial rate constants[2]. STD experiments were acquired with on and off resonance saturation at +0.1ppm and -40ppm respectively with 1048 scans each.

Solubility of compounds was determined in NMR buffer at pH 7.5 (see above) by integrating the signal of 250 µM maleic acid as an external standard relative to the integrals of the ligands. If the intended concentrations of 500 µM respective 2000 µM were reached the value is reported as > 500 µM or > 2000 µM. Compound 3 only reached a concentration of 1000 µM according to the QNMR experiment even though 2000 µM was intended, it's solubility is therefore reported as 1000µM.

Microscale Thermophoresis.

Fluorescence labeling of β-catenin$^{141-305}$ with the NT647 dye was achieved using the Monolith NT.115 Protein Labeling Kit RED-NHS according to the manufacturer's protocol (NanoTemper Technologies, Munich, Germany). The BCL9 peptide was used as positive control for assay development to achieve a reliably detectable change in the thermophoretic mobility (ΔF_{norm}). Experimental conditions were optimized to increase signal to noise ratio and assay window (buffer conditions, protein concentration, temperature gradient by infrared laser (IR) on time, capillaries). Final assay conditions were 50 nM NT647 labeled β-catenin$^{141-305}$ in PBS pH 6.8 supplemented with 100 mM $NaNO_3$, 0.5 mM TCEP and 0.05% Tween-20 at 25° C. Fragment screening detected

by MST was carried out as previously described[8,9]. In brief, 50 nM NT647-labeled β-catenin$^{141\text{-}305}$ was incubated with 500 μM compound and 1% (v/v) d6-DMSO at 298K. To achieve fully automated sample preparation and data collection an in house modified Monolith NT.015 was combined with a Hamilton Microlab Star pipetting robot in collaboration with Nanotemper Technologies[8,9]. Manual inspection of all MST traces was used to reject non-standard traces stemming from protein aggregation, bleaching, or fluorescence quenching. ΔF_{norm} values ($\Delta F_{norm}=F_{hot}/F_{cold}$) were calculated and the mean values of the duplicates were compared to the mean values of the DMSO negative control. Finally, fragment hits were identified by $\Delta\Delta F_{norm} \geq \Delta F_{norm}$(2sd DMSO) with $\Delta\Delta F_{norm} = |\Delta F_{norm}(\text{compound})-\Delta F_{norm}(\text{DMSO})|$. Protein integrity over the entire FBS was monitored by the BCL9 peptide positive control. For K_d determination a dilution series was pipetted using the fully automated set up using 200 mM DMSO stocks to achieve 2 mM compound at 1% DMSO as the highest concentration.

Surface Plasmon Resonance (SPR)

A Biacore T200 instrument was used for SPR analysis, β-catenin$^{141\text{-}305}$ was pre-diluted to a concentration of 0.1 mg/mL with 10 mM sodium acetate buffer, pH 5.0 and immobilized at a density of 6000 –7000 response units on flow cell 2 of a Biacore CM5 chip. Carbonic anhydraseII served as a reference protein on flow cell 3 and flow cell 1 was kept blank and used as the reference surface. A mixture of 20 mM Tris, pH 7.5, 150 mM NaCl, 0.5 mM TCEP, 0.005% Tween20, 2 % dimethyl sulfoxide was used as assay buffer. After pre-equilibration of the chip with 10 blank injections, the K_d measurements were performed by injecting a concentration series of analyte (9 concentrations, 1:1 dilutions). BCL9$^{347\text{-}392}$ served as a positive control. K_d values were determined by global fitting of the steady-state response for each of the 9 concentrations to a 1:1 interaction model using the Biacore T200 evaluation software. Each compound was injected three times and

average K_d values were calculated from the repeats. The reported data are the mean values of three independent experiments ± standard deviations.

Structural Analysis

Crystals of β-catenin[141-305] were obtained using the sitting drop vapor diffusion method. At 4° C 0.2 µL protein solution (13 mg/mL protein, 6.3 mM BI01450033) were mixed with 0.2µL reservoir solution containing 25% PEG 3350, 100 mM BIS-TRIS buffer pH 5.5 and 0.2 M Magnesium chloride. Brick-shaped crystals appeared after 24 hours and grew to a size of about 80 µM. Crystals were flash-frozen in liquid nitrogen in reservoir solution supplemented with 23% Ethylene glycol and 6% Di-Ethylene glycol. Diffraction data was collected in-house at a RIGAKU MICROMAX-003. Images were processed with autoPROC[10] and the structure was solved by molecular replacement using the previously solve structure (pdb ID: 3SLA) as model. Model building and refinement was performed with CCP4, COOT and autoBUSTER v.2.11.2. (http://www.globalphasing.com) using standard protocols[11,12]. The final model was analyzed with MolProbity revealing residues in 99.4% in Ramachandran preferred regions. Statistics for the data collection and refinement can be found in Supplementary Table 2 and stereo images of the binding modes (wall-eye stereo) with the refined 2Fo-Fc electron can be found in **Supplementary Fig. 7.**

Cell culture and transfection

Human embryonic kidney cells HEK293 (ATCC, CRL-1573, CVCL_0045) were cultivated in MEM Eagle with Earle's BSS w/NEAA, Pyr, w/o L-Gln medium (Lonza, BE12-662F) with Glutamax Supplement (Thermo Fisher Scientific, 35050061) with 10% FCS (GE Healthcare, SH30071.03). Transfections were performed with Lipofectamine LTX Reagent (Thermo Fisher, 15338100) according the manufacturer's instructions.

Luciferase reporter gene assay

For the dual luciferase assay, reverse transfection (0.4 x 10^5 cells, black 96 well plate) was performed using an active β-catenin control (S33A_S37A_T41A_S45A) and respective binding pocket mutants with co-transfection of a β-catenin/TCF-responsive TOPFlash luciferase reporter construct and constitutive active Renilla luciferase. 10 mM LiCl treatment was performed for 24h at 48 hours post transfection. Three days after transfection, the Dual-Glo® Luciferase Assay System (Promega, E2940) was applied following the manufacturer's instructions. Reporter activation obtained with transfection of 5 ng active β-catenin control was set to 100% as a reference.

Co-immunoprecipitation (Co-IP) and immunoblotting

For Co-IP, cells were transfected (5 x 10^6 cells/10 mL, 10 cm dish) with β-catenin constructs (4 µg) or pcDNA3.1 (4 µg) as a negative control and TCF4 (8 µg) or BCL9 (4 µg) 24 hours post seeding. Cells were lysed 48 h post transfection with 500 µL of ice-cold lysis buffer (22 mM Tris-HCl, 1.1% Triton, 275 mM NaCl, 11 mM EDTA, Halt Inhibitor 1:100, 10 mM DTT, 0.5 µL/mL Benzonase). 500 µg protein lysate were used for Co-IP. 50 µL of Anti-FLAG® M2 Magnetic Beads (Sigma, M8823) were added per sample and rotated over night at 4°C to allow the beads to bind Flag-tagged β-catenin and mutants. The next day, the beads were washed (3x 1 mL ice-cold PBS) using a magnetic rack, heated for 5 minutes at 95°C and 200 rpm for Western Blot preparation.

Western Blot

For Western Blot analysis, gel electrophoresis was performed with a 26 slot 4-12% Bis-Tris precast gel (Bio-Rad, 345-0125) and transferred to a 0.2 µM PVDF membrane (Bio-Rad, 170-4157) via the Trans-Blot Turbo Blotting system (Bio-Rad, 170-4155). For analysis of β-catenin expression

in Luciferase reporter gene assays, between 5 and 6.9 µg of protein per sample were used. For Co-IP analysis, 25 µg of pre-Co-IP samples were used as input control. Membranes were blocked with 4% milk powder/0.05% TBS-T. Anti-Flag antibody (Sigma, F1804, 1:1000) and anti-HA antibody (Roche, 12158167001, 1:2000) were used for detection. Anti-Actin (Sigma, A2066, 1:2000) served as a loading control.

Synthetic Chemistry

Compound **1** is commercially available at different vendors (e.g Aurora Fine Chemicals) and Compound **2** was purchased from InFarmatik Inc. Both compounds are members of the company's fragment library. Compound **3** is derived from internal research and is thus part of the compound library of Boehringer Ingelheim, the respective synthesis has been described in literature[13]. The synthetic sequences towards all other compounds as well as their respective characterizations are reported in the Supplementary Note. Reactions were carried out in standard commercially available glassware using standard synthetic chemistry methods until not noted otherwise. Reagents were obtained from commercial sources and used without additional purification.

List of abbreviations

AcOH	Acetic acid
MeCN	Acetonitrile
Boc	*tert*.butoxy carbonyl;
CAN	Cer(IV)-ammonium-nitrate
cHex	Cyclohexane
DAD	Diode array detector
DCM	Dichloromethane, CH_2Cl_2
Dppf	1,1'-Bis(diphenylphosphino)ferrocene
DIPEA	Diisopropylethyl amine

DME	1,2-Dimethoxyethane
DMF	*N*,*N*-Dimethylformamide
DMSO	Dimethylsulphoxide
EtOAc or EA	Ethyl acetate
EtOH	Ethanol
h	Hour(s)
HPLC	High performance liquid chromatography
HATU	O-(7-azabenzotriazol-1-yl)-1,1,3,3-tetramethyluronium hexafluorophosphate
HRMS	High resolution mass spectroscopy
INT	Intermediate
KOAc	Potassium acetate
LC	Liquid Chromatography
M	Molar (mol/L)
MeOH	Methanol
µL	Microliter
µm	Micrometer
Min	Minute(s)
mL	Milliliter
mm	Millimeter
MS	Mass spectrometry
MsCl	Methanesulfonyl chloride
nm	Nanometer
N	Normal
NBS	*N*-Bromosuccinimide
NMR	Nuclear magnetic resonance
PE	Petrolether
Pd_2dba_3	Tris(dibenzylideneacetone)dipalladium(0)
$Pd(dppf)Cl_2$	[1,1′-Bis(diphenylphosphino)ferrocene]dichloropalladium(II)
ppm	Parts per million

prot.	Protonated
RP	Reversed phase
Rt	Room temperature (20 to 25°C)
SM	Starting material
TEA	Triethylamine
TFA	Trifluoroacetic acid
THF	Tetrahydrofuran
t_R, Rt	Retention time [min]
XPhos	2-Dicyclohexylphosphino-2′,4′,6′-triisopropylbiphenyl

General Methods

Each synthetic transformation was monitored and confirmed by HPLC-MS. Commercial starting materials were used without further purification. Solvents used for reactions were of commercial “dry”- or “extra-dry” or “analytical” grade. All other solvents used were reagent grade. Air- and moisture-sensitive reactions were performed under dry nitrogen or argon atmosphere with dried glassware. Commercial starting materials were used without further purification. All solvents used for reactions were “dry”- or “extra-dry” or “analytical” grade. Other solvents were reagent grade. Preparative RP-HPLC purification was achieved on Agilent or Gilson systems using columns from Waters (Sunfire C18 OBD, 5 or 10 µm, 20 x 50 mm, 30 x5 0 mm or 50 x 150 mm; X-Bridge C18 OBD, 5 or 10 µm, 20 x 50, 30 x 50, or 50 x 150 mm) or YMC (Triart C18, 5 or 10 µm, 20 x 50 mm, or 30 x 50 mm). Compounds were eluted with MeCN/water gradients using either acidic (0.2% HCOOH or TFA) or basic water (5 mL 2 M NH_4HCO_3 + 2 mL NH_3 (32%) made up to 1 L with water).

Analytical NMR Spectroscopy:

NMR experiments were recorded on a Bruker Avance HD 500 MHz spectrometer equipped with a TCI cryoprobe at 298 K. Samples were dissolved in 600 μL DMSO-d_6 and TMS was added as an internal standard. 1D ^{1}H spectra were acquired with 30° excitation pulses and an interpulse delay of 4.2 sec with 64k data points and 20 ppm sweep width.

1D ^{13}C spectra were acquired with broadband composite pulse decoupling (WALTZ16) and an interpulse delay of 3.3 sec with 64 k data points and a sweep width of 240 ppm. Processing and analysis of 1D spectra was performed with Bruker Topspin 3.2 software. No zero filling was performed and spectra were manually integrated after automatic baseline correction. Chemical shifts are reported in ppm on the δ scale.

HSQC spectra were recorded on all samples to aid the interpretation of the data and to identify signals possibly hidden underneath solvent peaks. Spectra were acquired with sweep widths obtained by automatic sweep width detection from 1D reference spectra in the direct dimension with 1k datapoints and with 210 ppm and 256 datapoints in the indirect dimension.

Analytical LCMS:

Analytical LC/MS data were measured on an Agilent HPLC 1200 Series with Agilent LC/MSD SL detector using a Waters XBridge C18, 2.5 μm, 2.1 x 20 mm column. Solvent A [20 mM NH_4HCO_3/ NH_3 (pH 9)] and solvent B [acetonitrile HPLC grade] were used as eluent (additional settings: flow 1 mL.min^{-1}; injection volume 5 μL; column temp. 60 °C). Standard gradient: 0.00 min: 10% B; 0.00 – 1.50 min: 10% to 95% B; 1.50 – 2.00 min: 95% B; 2.00 – 2.10 min: 95% to 10% B.

Preparative RP-HPLC:

Preparative RP-HPLC was carried out on Agilent or Gilson systems using columns from Waters (Sunfire C18 OBD, 5 or 10 µm, 20x50 mm, 30x50 mm or 50x150 mm; X-Bridge C18 OBD, 5 or 10 µm, 20x50, 30x50, or 50x150 mm) or YMC (Triart C18, 5 or 10 µm, 20x50 mm, or 30x50 mm). Unless otherwise indicated compounds were eluted with MeCN/water gradients using either acidic (0.2% HCOOH or TFA) or basic water (5 ml 2 M NH_4HCO_3 + 2 ml NH_3 (32%) made up to 1 L with water).

Chiral Separation:

Chiral separation was performed with a Sepiatec SFC system. The following conditions were used, 20 x 250 mm IC column (Daicel) at a flow of 70ml/min, 40°C column temperature, a backpressure of 200 bar, and an isocratic eluent ratio of 40% methanol and 60% CO_2.

Synthesis of compounds 4 and 5

Enantiomers 4 and 5 were obtained from a chromatographic separation on a chiral phase of a racemic precursor and subsequent methylation of the single enantiomers as shown below:

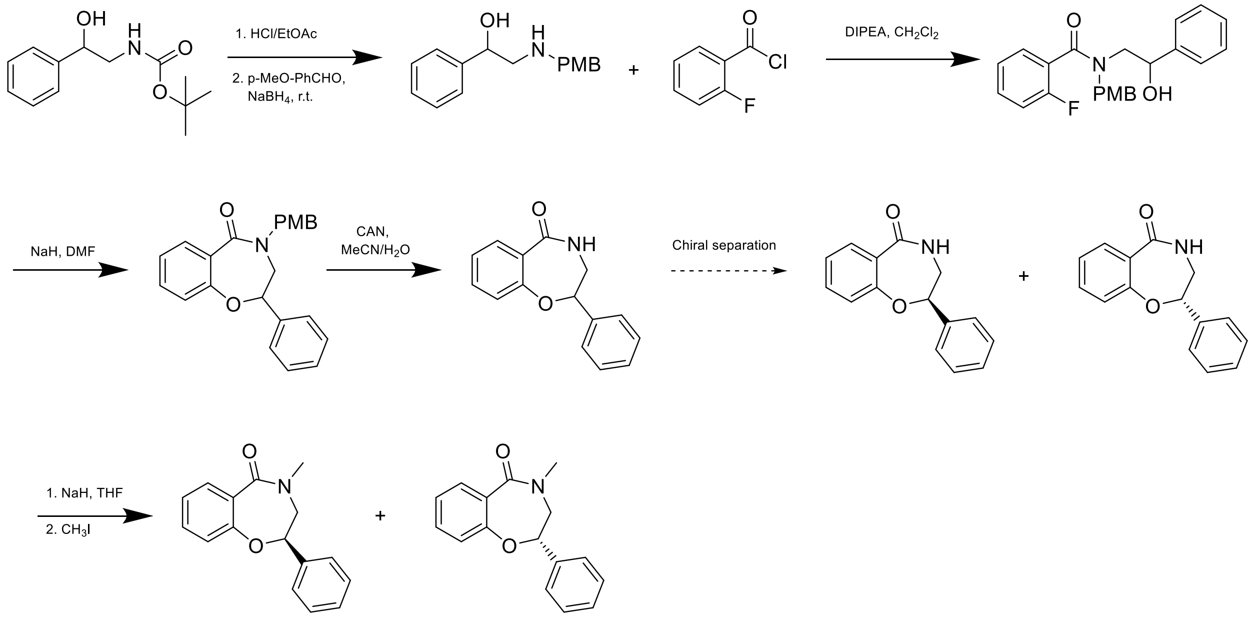

4 **5**

Synthesis of 2-(4-Methoxy-benzylamino)-1-phenyl-ethanol

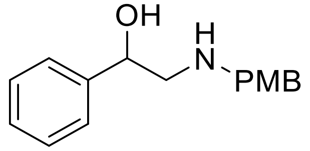

A solution of 10.00 g (42.0 mmol, 1.0 eq.) N-Boc-2-Hydroxy-2-Phenethylamine in 100 ml 1M HCl/EtOAc was stirred at room temperature overnight. The reaction mixture was filtered and the filter cake was dried under vacuum to afford the intermediate raw material that was dissolved in 100 ml dichloromethane. After the addition of 5.74 g (42.0 mmol, 1.0 eq.) para-Methoxy benzaldehyde the reaction mixture was stirred for 4 hours. Then 2.39 g (63.0 mmol, 1.5 eq) sodium borohydride were added and the mixture was kept stirring overnight. TLC showed complete conversion. To the reaction mixture was added 250 ml water and it was extracted three times with 50 mL EtOAc each. The combined organic layers were concentrated in vacuo and the remaining

crude product was purified by normal phase chromatography to obtain 6.00 g (23.0 mmol, 55%) 2-(4-Methoxy-benzylamino)-1-phenyl-ethanol.

HPLC-MS Rt = 1.20 min., [M+H]+: 258.

Synthesis of 4-(4-Methoxy-benzyl)-2-phenyl-3,4-dihydro-2H-benzo[f][1,4]oxazepin-5-one

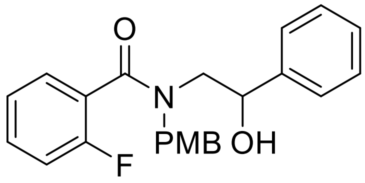

300 mg (1.89 mmol, 1.0 eq.) 2-Fluorobenzoylchloride were dissolved in 10 ml dichloromethane. Subsequently, 547 mg (2.08 mmol, 1.1 eq.) of 2-(4-Methoxy-benzylamino)-1-phenyl-ethanol and 655 µl (3.79 mmol, 2.0 eq.) of DIPEA were added and the mixture was stirred for 2 hours at r.t.

HPLC-MS analysis of the reaction mixture indicated full conversion. Water was added (50 ml) and the mixture was extracted with dichloromethane (3 x 50 mL). After the combined organic layers were dried over sodium sulfate the crude material was obtained after concentration under reduced pressure. The crude material (890 mg) was directly used in the subsequent step.

HPLC-MS Rt = 0.69 min., [M+H]+: 380.

Synthesis of 4-(4-Methoxy-benzyl)-2-phenyl-3,4-dihydro-2H-benzo[f][1,4]oxazepin-5-one

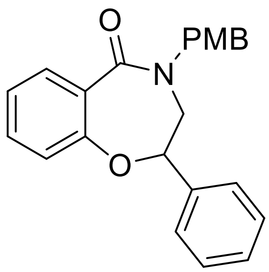

650 mg (1.7 mmol, 1.0 eq.) 2-Fluoro-N-(2-hydroxy-2-phenyl-ethyl)-N-(4-methoxy-benzyl)-benzamide was dissolved in 70 ml DMF and 190 mg sodium hydride (65% dispersion in oil) was added. The mixture was stirred overnight at r.t. Subsequent HPLC-MS analysis showed complete conversion. 150 ml dichloromethane were added to the mixture and it was washed with 3 x 50 ml water. The organic layer was dried over sodium sulfate and concentrated in vacuo. The obtained crude material was used for the next synthetic step without any further purification or characterisation.

HPLC-MS Rt = 0.77 min., [M+H]+: 360.

Synthesis of 2-Phenyl-3,4-dihydro-2H-benzo[f][1,4]oxazepin-5-one (rac)

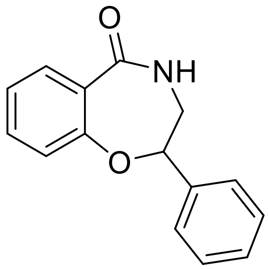

600 mg (1.67 mmol, 1.0 eq.) 4-(4-Methoxy-benzyl)-2-phenyl-3,4-dihydro-2H-benzo[f][1,4]oxazepin-5-one were dissolved in a a mixture of 18 ml acetonitrile and 6 ml water. 3.66 g (6.68 mmol, 4.0 eq.) Cer(IV)-ammonium nitrate were added and the mixture was stirred at r.t. for 30 mins. After HPLC-MS analysis had shown complete conversion dichloromethane

(50 ml) and water (100 ml) were added. The organic phase was separated and dried over sodium sulfate and concentrated under reduced pressure. The crude material was purified by RP-chromatography, and after lyophilisation of the respective fractions 79 mg (0.33 mmol, 20%) 2-Phenyl-3,4-dihydro-2H-benzo[f][1,4]oxazepin-5-one (rac.) were obtained.

Chiral Separation:

Racemic 2-Phenyl-3,4-dihydro-2H-benzo[f][1,4]oxazepin-5-one was separated by chiral chromatography on an SFC system with the conditions described in the general methods section above. (R)-2-Phenyl-3,4-dihydro-2H-benzo[f][1,4]oxazepin-5-one was the first eluting compound with a Rt of 1.07 min and was obtained with an e.e. value of >95%. (S)-2-Phenyl-3,4-dihydro-2H-benzo[f][1,4]oxazepin-5-one eluted second with an Rt value of 1.43 min and was obtained with an e.e. value of >98%

Compound 4: Synthesis of (R)-4-Methyl-2-phenyl-3,4-dihydro-2H-benzo[f][1,4]oxazepin-5-one 4

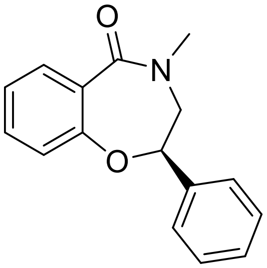

15.0 mg (0.063 mmol, 1 eq.) (R)-2-Phenyl-3,4-dihydro-2H-benzo[f][1,4]oxazepin-5-one were dissolved in 1 ml THF and 4.5 mg (0.075 mmol, 1.2 eq, 40% dispersion in oil) sodium hydride were added. The reaction mixture was stirred for 1 hour at r.t. After the addition of 9.9 mg (0.069 mmol, 1.1 eq.) iodomethane the reaction mixture was stirred overnight. HPLC-MS analysis showed complete conversion. The mixture was directly loaded on isolute and subjected to normal

phase silica gel based chromatography (DCM/MeOH). After evaporation of the respective fractions 15.0 mg (0.059 mmol, 95%) compound **4** were obtained.

HPLC-MS Rt = 1.15 min., [M+H]+: 254

^{1}H NMR (DMSO-d$_6$, 500 MHz) δ 7.64 (dd, 1H, *J*=1.6, 7.9 Hz), 7.49 (dt, 1H, *J*=1.6, 7.7 Hz), 7.4-7.4 (range, 4H), 7.3-7.4 (m, 1H), 7.22 (t, 1H, *J*=7.6 Hz), 7.04 (d, 1H, *J*=8.2 Hz), 5.66 (dd, 1H, *J*=3.5, 6.6 Hz), 3.6-3.9 (m, 2H), 2.90 (s, 3H)

^{13}C NMR (DMSO-d$_6$, 126 MHz) δ 167.4, 153.1, 139.3, 132.6, 130.3, 128.5, 128.1, 128.1, 126.2, 123.6, 122.2, 84.4, 52.7, 35.0

Compound 5: Synthesis of (S)-4-Methyl-2-phenyl-3,4-dihydro-2H-benzo[f][1,4]oxazepin-5-one 5

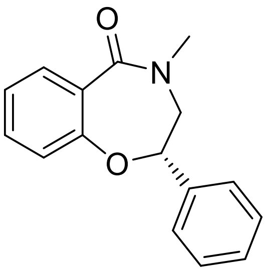

19.0 mg (0.079 mmol, 1 eq.) (S)-2-Phenyl-3,4-dihydro-2H-benzo[f][1,4]oxazepin-5-one were dissolved in 1 ml THF and 5.7 mg (0.095 mmol, 1.2 eq, 40% dispersion in oil) sodium hydride were added. The reaction mixture was stirred for 1 hour at r.t. After the addition of 12.5 mg (0.087 mmol, 1.1 eq.) iodomethane the reaction mixture was stirred overnight. HPLC-MS analysis showed complete conversion. The mixture was poured into a 1 M aqueous solution of NaOH and extracted with dichloromethane several times. The combined organic layers were dried over magnesium sulfate and concentrated under reduced pressure. The crude material was loaded on

isolute and subjected to normal phase silica gel based chromatography (DCM/MeOH). After evaporation of the respective fractions 9.5 mg (0.037 mmol, 47%) compound **5** were obtained.

HPLC-MS Rt = 1.15 min., [M+H]+: 254

^{1}H NMR (DMSO-d_6, 500 MHz) δ 7.64 (dd, 1H, *J*=1.6, 7.9 Hz), 7.49 (dt, 1H, *J*=1.6, 7.7 Hz), 7.4-7.4 (range, 4H), 7.3-7.4 (m, 1H), 7.22 (t, 1H, *J*=7.6 Hz), 7.04 (d, 1H, *J*=8.2 Hz), 5.66 (dd, 1H, *J*=3.5, 6.6 Hz), 3.6-3.9 (m, 2H), 2.90 (s, 3H)

^{13}C NMR (DMSO-d_6, 126 MHz) δ 167.4, 153.1, 139.3, 132.6, 130.3, 128.5, 128.1, 128.1, 126.2, 123.6, 122.2, 84.4, 52.7, 35.0

Synthesis of compounds 6 and 7

Enantiomers 6 and 7 were obtained from a chromatographic chiral separation on a chiral phase of the racemate accessible via the route shown below.

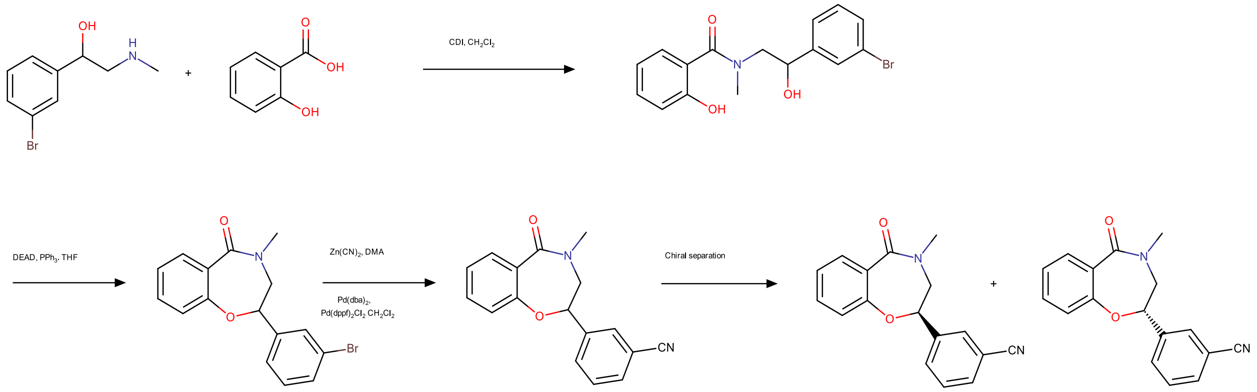

Synthesis of N-[2-(3-Bromo-phenyl)-2-hydroxy-ethyl]-2-hydroxy-N-methyl-benzamide

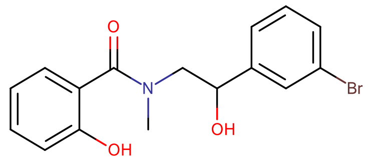

62.7 mg (0.45 mmol, 1.1 eq) salicylic acid was dissolved in dichloromethane (3 mL) and after addition of 100.4 mg (0.62 mmol, 1.5 eq) DCC the mixture was stirred for 2 hours at r.t. After cooling to 0 °C 100 mg (0.41 mmol, 1.0 eq) 1-(3-Bromophenyl)-2-(Methylamino)-ethan-1-ol was added and the reaction mixture stirred for 3 hours until HPLC-MS reaction control showed significant consumption of the starting material. After concentration under reduced pressure the raw material was purified by RP-chromatography to deliver 32 mg (0.09 mmol, 22%) of the desired benzamide.

HPLC-MS Rt = 1.05 min., [M+H]+: 254.

^{1}H NMR (400 MHz, DMSO-d_6, 25ºC): δ = 9.61-9.96 (m, 2H), 7.58 (br s, 1H), 7.27-7.51 (m, 5H), 7.16-7.25 (m, 4H), 7.12 (br s, 1H), 7.00 (br s, 2H), 6.75-6.88 (m, 5H), 5.62-5.78 (m, 2H), 4.89 (br s, 1H), 4.59-4.70 (m, 1H), 3.53-3.61 (m, 1H), 3.19-3.28 (m, 1H), 3.01 (br s, 3H), 2.74 ppm (br s, 3H). Rotamers, ratio ~ 1:1, double set of signals

Synthesis of 2-(3-Bromo-phenyl)-4-methyl-3,4-dihydro-2H-benzo[f][1,4]oxazepin-5-one

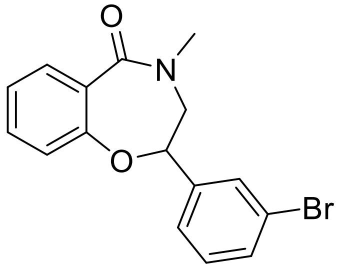

298 mg (1.08 mmol, 1.1 eq.) Triphenylphosphine were dissolved in 20 ml THF and 218.3 mg (1.08 mmol, 1.0 eq.) DIAD were added at 0 °C. After 5 min. of stirring 378 mg (1.08 mmol, 1.0 eq) N-[2-(3-Bromo-phenyl)-2-hydroxy-ethyl]-2-hydroxy-N-methyl-benzamide dissolved in 1 ml THF were added dropwise. After 16 hours HPLC-MS analysis showed complete conversion. The reaction mixture was loaded onto isolute and purified by RP-chromatography to yield 92 mg (0.28 mmol, 51 %) racemic 2-(3-Bromo-phenyl)-4-methyl-3,4-dihydro-2H-benzo[f][1,4]oxazepin -5-one.

HPLC-MS Rt = 0.67 min., [M+H]+: 332/334.

^{1}H NMR (400 MHz, DMSO-d$_6$, 25ºC): δ = 7.62-7.66 (m, 2H), 7.58 (dt, *J* = 7.7, 1.6 Hz, 1H), 7.51 (td, *J* = 7.7, 1.8 Hz, 1H), 7.44-7.47 (m, 1H), 7.37-7.42 (m, 1H), 7.24 (td, *J* = 7.5, 1.1 Hz, 1H), 7.07 (dd, *J* = 8.2, 0.9 Hz, 1H), 3.66-3.84 (m, 2H), 2.91 ppm (s, 3H)

Synthesis of 3-(4-Methyl-5-oxo-2,3,4,5-tetrahydro-benzo[f][1,4]oxazepin-2-yl)-benzonitrile

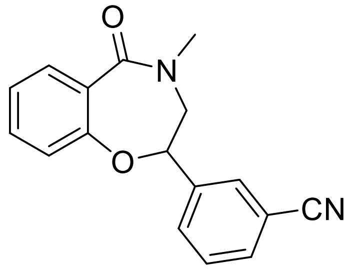

92.0 mg (0.28 mmol, 1 eq.) 2-(3-Bromo-phenyl)-4-methyl-3,4-dihydro-2H-benzo[f][1,4]oxazepin-5-one were dissolved in DMA (2 mL). Under a nitrogen atmosphere sequentially 23.3 mg (0.028 mmol, 0.1 eq) Pd(dppf)$_2$Cl$_2$·CH$_2$Cl$_2$, 15.9 mg (0.028 mmol, 0.1 eq.) Pd(dba)2 and 9.1 mg

(0.14 mmol, 0.5 eq.) zinc powder were added and the mixture was irradiated in a microwave at 150 °C for 12 hours. HPLC-MS analysis showed complete conversion.

The mixture was poured into water and extracted three times with each 20 ml dichloromethane. After combination of the organic phases they were dried over magnesium sulfate and concentrated under reduced pressure. The crude product was purified via RP-chromatography to yield 15.0 mg (0.05 mmol, 20%) racemic 3-(4-Methyl-5-oxo-2,3,4,5-tetrahydro-benzo[f][1,4]oxazepin-2-yl)-benzonitrile.

HPLC-MS Rt = 1.06 min., [M+H]+: 279.

Chiral separation:

Racemic 3-(4-Methyl-5-oxo-2,3,4,5-tetrahydro-benzo[f][1,4]oxazepin-2-yl)-benzonitrile was separated by chiral chromatography on an SFC system based on the method described in the general procedures section above. Compound 6 eluted first with a Rt of 1.12 min and was obtained with an e.e. value of >97%. Compound 7 eluted second with a Rt of 1.34 min and was obtained with an e.e. value of >98%

Analytical data of enantiomers 6 and 7:

Compound 6: (R)-3-(4-Methyl-5-oxo-2,3,4,5-tetrahydro-benzo[f][1,4]oxazepin-2-yl)-benzonitrile

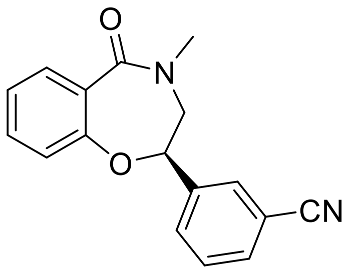

HPLC-MS Rt = 1.06 min., [M+H]+: 279

^{1}H NMR (DMSO-d_6, 500 MHz) δ 7.89 (s, 1H), 7.86 (d, 1H, *J*=7.6 Hz), 7.80 (d, 1H, *J*=7.9 Hz), 7.6-7.7 (m, 2H), 7.51 (dt, 1H, *J*=1.9, 7.7 Hz), 7.25 (t, 1H, *J*=7.4 Hz), 7.09 (d, 1H, *J*=8.2 Hz), 5.73 (dd, 1H, *J*=3.5, 6.0 Hz), 3.7-3.9 (m, 2H), 2.88 (s, 3H)

^{13}C NMR (DMSO-d_6, 125 MHz) δ 167.4, 152.8, 140.8, 132.8, 131.9, 131.1, 130.3, 129.9, 129.8, 128.0, 123.9, 122.0, 118.6, 111.4, 83.4, 52.2, 35.1

Compound 7: (S)-3-(4-Methyl-5-oxo-2,3,4,5-tetrahydro-benzo[f][1,4]oxazepin-2-yl)-benzonitrile

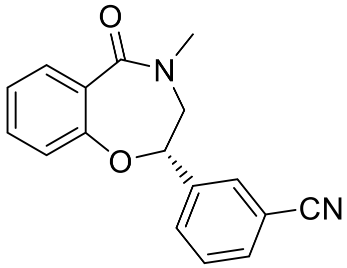

HPLC-MS Rt = 1.06 min., [M+H]+: 279.

^{1}H NMR (DMSO-d_6, 500 MHz) δ 7.89 (s, 1H), 7.86 (d, 1H, *J*=7.6 Hz), 7.80 (d, 1H, *J*=7.9 Hz), 7.6-7.7 (m, 2H), 7.51 (dt, 1H, *J*=1.9, 7.7 Hz), 7.25 (t, 1H, *J*=7.4 Hz), 7.09 (d, 1H, *J*=8.2 Hz), 5.73 (dd, 1H, *J*=3.5, 6.0 Hz), 3.7-3.9 (m, 2H), 2.88 (s, 3H)

^{13}C NMR (DMSO-d_6, 125 MHz) δ 167.4, 152.8, 140.8, 132.8, 131.9, 131.1, 130.3, 129.9, 129.8, 128.0, 123.9, 122.0, 118.6, 111.4, 83.4, 52.2, 35.1

Supplementary References

1. de la Roche, M. et al. An intrinsically labile α-helix abutting the BCL9-binding site of β-catenin is required for its inhibition by carnosic acid. *Nature Communications* **3**, 680 (2012).
2. Mayer, M. & James, T.L. NMR-Based Characterization of Phenothiazines as a RNA Binding Scaffold. *Journal of the American Chemical Society* **126**, 4453-4460 (2004).
3. Alfred, R. & Hans, S. Automation of Biomolecular NMR Screening. *Current Topics in Medicinal Chemistry* **3**, 55-67 (2003).
4. Pervushin, K.V., Wider, G. & Wüthrich, K. Single Transition-to-single Transition Polarization Transfer (ST2-PT) in [15N,1H]-TROSY. *Journal of Biomolecular NMR* **12**, 345-348 (1998).
5. Peng, C., Unger, S.W., Filipp, F.V., Sattler, M. & Szalma, S. Automated evaluation of chemical shift perturbation spectra: New approaches to quantitative analysis of receptor-ligand interaction NMR spectra. *J Biomol NMR* **29**, 491-504 (2004).
6. Piotto, M., Saudek, V. & Sklenář, V. Gradient-tailored excitation for single-quantum NMR spectroscopy of aqueous solutions. *Journal of Biomolecular NMR* **2**, 661-665 (1992).
7. Geist, L. et al. Direct NMR Probing of Hydration Shells of Protein Ligand Interfaces and Its Application to Drug Design. *Journal of Medicinal Chemistry* **60**, 8708-8715 (2017).
8. Martin, L.J. et al. Structure-Based Design of an in Vivo Active Selective BRD9 Inhibitor. *Journal of medicinal chemistry* **59**, 4462-4475 (2016).
9. Kessler, D. et al. Drugging an undruggable pocket on KRAS. *Proceedings of the National Academy of Sciences* **116**, 15823-15829 (2019).
10. Vonrhein, C. et al. Data processing and analysis with the autoPROC toolbox. *Acta Crystallographica Section D* **67**, 293-302 (2011).
11. Emsley, P., Lohkamp, B., Scott, W.G. & Cowtan, K. Features and development of Coot. *Acta Crystallographica Section D* **66**, 486-501 (2010).
12. Collaborative. The CCP4 suite: programs for protein crystallography. *Acta Crystallographica Section D* **50**, 760-763 (1994).
13. Weber, K.H. Synthese und Eigenschaften von 4-Alkyl-2-phenyl-3,4-dihydro-5H-1,4-benzodiazepin-5-onen. *Archiv der Pharmazie* **302**, 584-590 (1969).
